# Nociceptive Nerve‐Derived CGRP Exacerbates Uterine Fibrogenesis in Adenomyosis by Promoting CD140b^+^ CD146^+^ Fibroblast Differentiation

**DOI:** 10.1002/advs.202507128

**Published:** 2025-09-23

**Authors:** Zi Ye, Anning Zhao, Xia Li, Yanqing Hao, Dong Huang, Jianmin Chen, Tiantian Li, Yangyang Dai, Wenchao Sun, Lie Ma, Songying Zhang, Liaobing Xin

**Affiliations:** ^1^ Assisted Reproduction Unit Department of Obstetrics and Gynecology Sir Run Run Shaw Hospital School of Medicine Zhejiang University Hangzhou 310016 China; ^2^ Zhejiang Provincial Clinical Research Center for Reproductive Health and Disease Hangzhou 310016 China; ^3^ Zhejiang Key Laboratory of Precise Protection and Promotion of Fertility Hangzhou 310016 China; ^4^ MOE Key Laboratory of Macromolecular Synthesis and Functionalization Department of Polymer Science and Engineering Zhejiang University Hangzhou 310058 China; ^5^ Department of Reproductive Endocrinology Hangzhou Women's Hospital Hangzhou 310008 China

**Keywords:** adenomyosis, CD140b^+^ CD146^+^ fibroblasts, CGRP, fibrogenesis, nociceptive nerve

## Abstract

Progressive dysmenorrhea and extensive fibrosis within the myometrium are hallmark features of adenomyosis (AM). Approximately 80% of patients with AM experience secondary dysmenorrhea, which occurs more frequently in AM than in other gynecological disorders. Because nociceptive nerves mediate dysmenorrhea, their contribution to AM‐associated fibrosis is investigated. These results demonstrate overexpression of calcitonin gene‐related peptide (CGRP)+ nociceptive nerves within fibrotic lesions in both AM patients and murine models; nociceptive nerve ablation reduces uterine fibrosis in mice with induced AM. CGRP, secreted by nociceptive nerves, drives receptor activity‐modifying protein 1 (RAMP1) high‐expressing (RAMP1^hi^) CD140b^+^ CD146^+^ fibroblasts in AM lesions toward an extracellular matrix deposition subtype through activation of the extracellular signal‐regulated kinase pathway. Treatment of mice with AM using rimegepant, a United States Food and Drug Administration‐approved drug that blocks CGRP/RAMP1 signaling, alleviated progression of AM‐associated fibrosis and promoted fertility restoration. This study identifies a previously unrecognized nerve‐fibroblast crosstalk mechanism and provides a potential nonhormonal therapeutic strategy for AM treatment, particularly in women of reproductive age.

## Graphical abstract

This study investigates adenomyosis fibrosis in relation to nociceptive nerves. Nociceptive nerve‐secreted CGRP promotes RAMP1^hi^ CD140b^+^ CD146^+^ fibroblast differentiation into myofibroblasts through ERK activation, causing severe fibrosis. Rimegepant‐induced CGRP/RAMP1 signaling blockade alleviates fibrosis and restores fertility in a mouse model. This work provides a novel perspective regarding fibrotic mechanisms of adenomyosis and highlights the potential of nonhormonal therapeutic strategies.

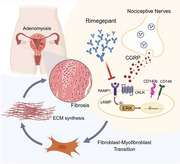

## 1 Introduction

Adenomyosis (AM) is a common, benign, and chronic gynecological disorder that affects 7–23% of women of childbearing age.^[^ ^1^, ^2^ ^]^ AM is characterized by the aberrant presence of endometrial glands and/or stroma within the myometrium, the muscular wall of the uterus. Ectopic endometrial glands and stroma retain their responsiveness to estrogen but fail to shed during menstruation, resulting in focal inflammation and repeated cycles of tissue injury and repair.^[^ ^3^ ^]^ The lesions undergo epithelial–mesenchymal transition, fibroblast‐to‐myofibroblast transdifferentiation, and smooth muscle metaplasia.^[^ ^4^, ^5^ ^]^ These processes lead to progressive fibrosis of the myometrium. Increased fibrosis within the lesion also damages the adjacent endometrial–myometrial interface and contributes to fibrosis of the eutopic endometrium.^[^ ^4^, ^6^ ^]^

In patients with fertility intentions and AM complications, AM‐induced fibrosis of the myometrium exerts a deleterious effect.^[^ ^7^ ^]^ The adverse myometrial environment is associated with substantially elevated risks of recurrent implantation failure, miscarriage, preterm labor, stillbirth, and multiple obstetric complications.^[^ ^8^ ^]^

Current treatment options for AM patients who wish to preserve fertility include uterine‐sparing focal lesion excision, hormone therapies such as gonadotropin‐releasing hormone agonist, dienogest, and the levonorgestrel‐releasing intrauterine system, along with assisted reproductive technologies after surgical and pharmacological pretreatment.^[^ ^9^, ^10^ ^]^ However, hormone therapy is associated with side effects that include perimenopausal syndrome, depression, bone loss, hot flashes, sweating, breakthrough bleeding, and cardiovascular risks.^[^ ^11^, ^12^ ^]^ Consequently, these medications are unsuitable for long‐term use, and some patients respond poorly to them. Thus, there is a critical need for nonhormonal treatments for AM that do not disrupt the hypothalamic–pituitary–ovarian axis.

Dysmenorrhea is the most common AM‐related complaint, occurring in ≈80% of women.^[^ ^13^, ^14^, ^15^ ^]^ Ectopic endometrial tissue in the myometrium is in close contact with and hyperinnervated by nerve fibers,^[^ ^16^ ^]^ and nociceptive nerves have been detected in ectopic lesions in women with AM.^[^ ^17^, ^18^ ^]^ Moreover, in endometriosis, a disease that shares similarities with AM, nociceptive nerve denervation led to a significantly greater reduction in lesion weight relative to sympathetic denervation in a murine model.^[^ ^19^, ^20^ ^]^ Calcitonin gene‐related peptide (CGRP) is a neuropeptide mainly released from the smallest‐diameter unmyelinated nociceptive neurons located in the dorsal root ganglia (DRG).^[^ ^21^ ^]^ CGRP signals through the calcitonin receptor‐like receptor in conjunction with receptor activity‐modifying protein 1 (RAMP1); it participates in multiple physiological and pathophysiological processes, including wound healing, inflammatory responses, extracellular matrix homeostasis, and energy metabolism.^[^ ^22^ ^]^ A previous work demonstrated that *Ramp1* knockout mice exhibited reduced formation of blood and lymphatic vessels in endometrial lesions, indicating that RAMP1 signaling is important for the establishment of endometriosis lesions.^[^ ^23^ ^]^ Additionally, a recent study demonstrated that CGRP mediates neuro‐immune communication and accelerates lesion growth in endometriosis.^[^ ^24^ ^]^ Compared with endometriosis, however, few studies have investigated nociceptive nerve fibers in AM.

Although the precise mechanism of AM remains unclear, there is speculation that it involves endometrial invasion, metaplasia of embryonic pluripotent Müllerian remnants, or differentiation from stem cells.^[^ ^1^, ^25^ ^]^ A considerable proportion of stem/progenitor cells, including stage‐specific embryonic antigen (SSEA1)^+^ epithelial cells, CD140b^+^ CD146^+^ cells, and sushi domain‐containing 2 (SUDS2)^+^ mesenchymal stem cells, is present in the endometrium.^[^ ^26^, ^27^ ^]^ In diseases such as osteoarthritis, skin regeneration, and pulmonary fibrosis, the nervous system plays a key role in regulating stem cell behavior, thus influencing tissue morphogenesis, regeneration, and disease progression.^[^ ^28^, ^29^ ^]^ A clinical retrospective study demonstrated a significant positive correlation between dysmenorrhea severity and lesion hardness in patients with AM.^[^ ^30^ ^]^ These findings imply the existence of a nerve–fibroblast interaction niche in AM lesions. Nevertheless, the interaction between fibroblasts and sensory nerves in AM lesions has not been investigated.

In this study, we detected a strong relationship between excessive nociceptive nerves and AM‐associated fibrosis. Single‐cell RNA sequencing (scRNA‐seq) revealed a correlation between CGRP^+^ nociceptive nerves and differentiation of CD140b^+^ CD146^+^ fibroblasts. In vitro functional assays of RAMP1 high‐expressing (RAMP1^hi^) CD140b^+^ CD146^+^ fibroblasts from AM lesions validated the phenotypic switch function and the underlying mechanism mediated by nociceptive nerve‐derived CGRP. In vivo pharmacological inhibition confirmed the efficacy of rimegepant, a RAMP1 antagonist, which resulted in pain relief, fibrosis remission, and fertility restoration in AM model mice. Collectively, these findings identify potential therapeutic approaches for the development of nonhormonal, fertility‐sparing treatments for women of childbearing age with AM.

## 2 Results

### 2.1 Hyperexpression of CGRP^+^ Nociceptive Nerves was Observed in AM Lesions Accompanied by Focal Fibrosis in Patient Samples and a Murine Model

**Figure 1:**
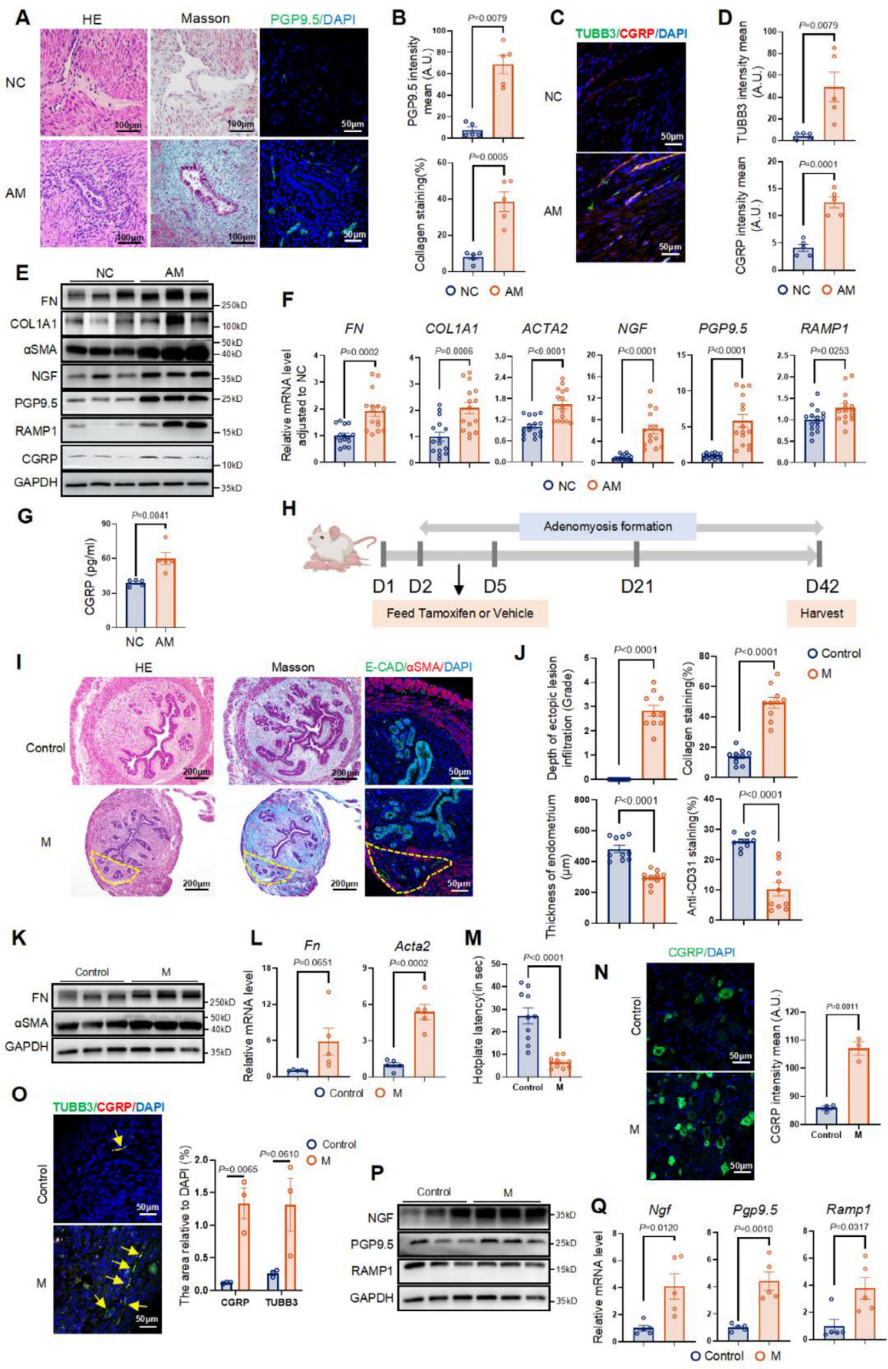
Hyperexpression of CGRP^+^ nociceptive nerve was observed in AM lesions accompanied by focal fibrosis in patient samples and a murine model. A) Representative images of Hematoxylin and eosin (H&E) and the corresponding horizon of IF stained with PGP9.5 (green) and Masson trichrome staining images in the myometrium from NC and AM. Scale bar, left, 100 µm; middle, 100 µm; right, 50 µm. B) Semi‐quantification and statistical analysis of PGP9.5 intensity and collagen areas. *n* = 5 patients per group. C,D) Representative images (C) and quantification (D) of IF with anti‐TUBB3 (green), CGRP (red), and nucleus (DAPI, Blue) in the myometrium from NC and AM. *n* = 5 patients per group. Scale bar, 50 µm. E) Western blot (WB) detection of FN, COLA1, αSMA, PGP9.5, NGF, RAMP1, and CGRP. *n* = 5 per group, three independent biological replicates on bands. F) The relative mRNA levels of *FN*, *COLA1*, *ACTA2* (gene name of αSMA), *PGP9.5*, *NGF*, and *RAMP1* in NC and AM. *n* = 15 patients per group. G) Quantitative analysis of ELISA assay for CGRP concentration in ectopic lesions from AM patients and normal myometrium from NC individuals. *n* = 5 patients per group. H) Schematic diagram of the establishment of AM model mice. I) H&E, Masson trichrome staining of mice uteri at Day 42 and the corresponding horizon of multiple IF staining of E‐Cadherin (E‐CAD, green, a marker for epithelial cells), αSMA(red, a marker for smooth muscle cells), and nucleus (DAPI, Blue) in Control and M groups. The yellow dotted line shows the location of the ectopic lesion. Scale bar, left, 200  µm; middle, 200 µm; right, 50 µm. J) Measurement of the depth of the ectopic lesion infiltration, eutopic endometrium, semiquantitative analysis of the collagen areas by Masson trichrome staining, and anti‐CD31 staining by IHC between the Control and M groups. *n* = 10 mice per group. K) WB analysis of FN, αSMA in Control and M. *n* = 5 per group. Three independent biological replicates on bands. L) The relative expression levels of *Fn*, *Acta2* in Control and M groups detected by qPCR. *n* = 5 mice per group. M) Hotplate latency measured between the Control and M groups. *n* = 10 per group. N) Representative images (left) and quantification (right) of IF co‐staining of CGRP (green) and nucleus (DAPI, Blue) in the mice DRG between the Control and M groups. *n* = 3 mice per group. Scale bar, 50 µm. O) Representative images (left) and quantification (right) of multiple IF staining of TUBB3 (green), CGRP (red), and nucleus (DAPI, Blue) in the uteri of mice. Yellow arrows: nociceptive nerves. *n* = 3 mice per group. Scale bar, 50 µm. P) WB analysis of NGF, PGP9.5 and RAMP1 in Control and M groups. *n* = 5 per group. Three independent biological replicates on bands. Q) The relative expression levels of *Ngf*, *Pgp9.5*, and *Ramp1* in Control and M groups were detected by qPCR. *n* = 5 mice per group. Results in B, D, F, G, J, L, M, N, O, Q are expressed as the mean ± SEM, Student's t‐test. *p*‐value was shown in plots. NC: negative control group; AM: adenomyosis group; Control: control group of mice; M: mice with induced adenomyosis.

We first investigated the relationship between nerve fiber density and fibrosis in the uterine myometrium of patients with AM. In contrast to the myometrium of the negative control group (NC, *n* = 5), the myometrium of the AM group (AM, *n* = 5) exhibited pathological invasion of glands and stroma (**Figure** **1**A; Figure S1, Supporting Information). Immunofluorescence (IF) analysis of full‐thickness uterine samples revealed increased nerve fibers in the basal layer and junctional zone in the AM group compared with the NC group (Figure S1, Supporting Information). Around ectopic lesions, excessive collagen deposition was observed by Masson trichrome staining; enriched nerve fibers stained with the marker protein gene product 9.5 (PGP9.5, also known as ubiquitin C‐terminal hydrolase L1) exhibited colocalization (Figure 1A,B). Sensory nerve density was then examined by co‐staining for the nerve fiber marker tubulin beta 3 class III (TUBB3) and the nociceptive nerve marker CGRP. The results demonstrated that CGRP^+^ nociceptive nerves were enriched in AM lesions (Figure 1C,D).

We next evaluated the expression patterns of fibrosis‐related proteins fibronectin (FN) and collagen type I alpha 1 chain (COL1A1); the myofibroblast marker alpha smooth muscle actin (αSMA); and nerve‐related proteins, including nerve growth factor (NGF), PGP9.5, CGRP, and its receptor RAMP1. Western blot (WB) analysis revealed increased expression of these proteins in AM lesions compared with NC samples (Figure 1E). Consistent with the WB results, quantitative polymerase chain reaction (qPCR) analysis showed elevated expression of the genes encoding *FN*, *COL1A1*, *αSMA* (gene name: *ACTA2*), *NGF*, *PGP9.5*, and *RAMP1* in AM lesions (Figure 1F). These results indicated that the fibrotic state in disease progression was accompanied by neurogenesis signaling and an increased density of nociceptive nerve fibers. Enzyme‐linked immunosorbent assay (ELISA) and immunohistochemistry (IHC) (NC, *n* = 5; AM, *n* = 5) investigations confirmed that CGRP expression was elevated in AM lesions compared with normal myometrium (Figure 1G; Figure S2, Supporting Information). Thus, we concluded that CGRP^+^ nociceptive nerves may interact with ectopic lesions and promote fibrosis progression in AM.

The above phenotype was also observed in the tamoxifen‐induced adenomyosis mouse model (M). The study design for animal experiments is shown in Figure 1H. Uteri were harvested from mice (Control, *n* = 10; M, *n* = 10) at 6 weeks, in accordance with a previous study.^[^ ^31^ ^]^ IF staining demonstrated the expression of E‐cadherin (an epithelial cell marker) in the disrupted myometrium of the M group, whereas the Control group exhibited normal myometrial and endometrial growth patterns (Figure 1I, J; Figure S3, Supporting Information). These results confirmed the successful establishment of the AM model. A substantial increase in collagen deposition was observed in uterine tissue, indicating AM‐induced enhancement of fibrotic remodeling (Figure 1I,J). Inflammation in the myometrium and disruption of the endometrial‐myometrial interface also contributed to pathological changes in the eutopic endometrium. Previous studies and our findings suggested that both ectopic lesions and eutopic endometrium exhibit immunological deregulation, including increases in lymphocyte and macrophage populations and elevated proinflammatory cytokines (Figure S4, Supporting Information),^[^ ^32^ ^]^ which result in dysfunction of the eutopic endometrium (e.g., fibrosis, endometrial thinning, and reduced blood supply).^[^ ^6^ ^]^ To characterize these changes in AM model mice, we measured endometrial thickness and vascular density. Compared with the Control group, the M group showed significant reductions in endometrial thickness (*p *< 0.0001) and blood supply (*p *< 0.0001) (Figure 1I,J; Figure S5, Supporting Information). Protein and gene expression levels of FN and αSMA were also elevated in the M group (Figure 1K,L). Consistent with previous studies,^[^ ^19^ ^]^ mice with AM exhibited thermal hyperalgesia, as reflected by shorter hotplate latencies (Figure 1M).

Unlike human samples, CGRP protein was not detected by WB in the uterus of AM mice (data not shown). This absence may be attributed to the short half‐life of CGRP in tissue after release and the large difference in ectopic lesion volume between humans and mice.^[^ ^22^ ^]^ However, more CGRP^+^ neurons were detected in the DRG of the M group (*n* = 3) compared with the Control group (*n* = 3) (Figure 1N). The uterus from AM mice (*n* = 3) also exhibited elevated levels of CGRP^+^ nociceptive nerve fibers accompanied by higher protein and mRNA expression levels of NGF and PGP9.5 compared with the Control group (Figure 1O–Q). An unanticipated finding was that RAMP1 protein expression was less evident in AM mice than in human samples. A possible explanation for this discrepancy is the difference in sample collection methodology: ectopic lesions in the myometrium were analyzed in humans, whereas full‐thickness uterine samples were analyzed in mice. Given that RAMP1 is also distributed in the endometrial stroma,^[^ ^33^ ^]^ the inclusion of full‐thickness uterine tissue from mice may have partially masked the differences.

### 2.2 Nociceptive Nerve Ablation Reduced Uterine Fibrosis in Mice with Induced AM

**Figure 2:**
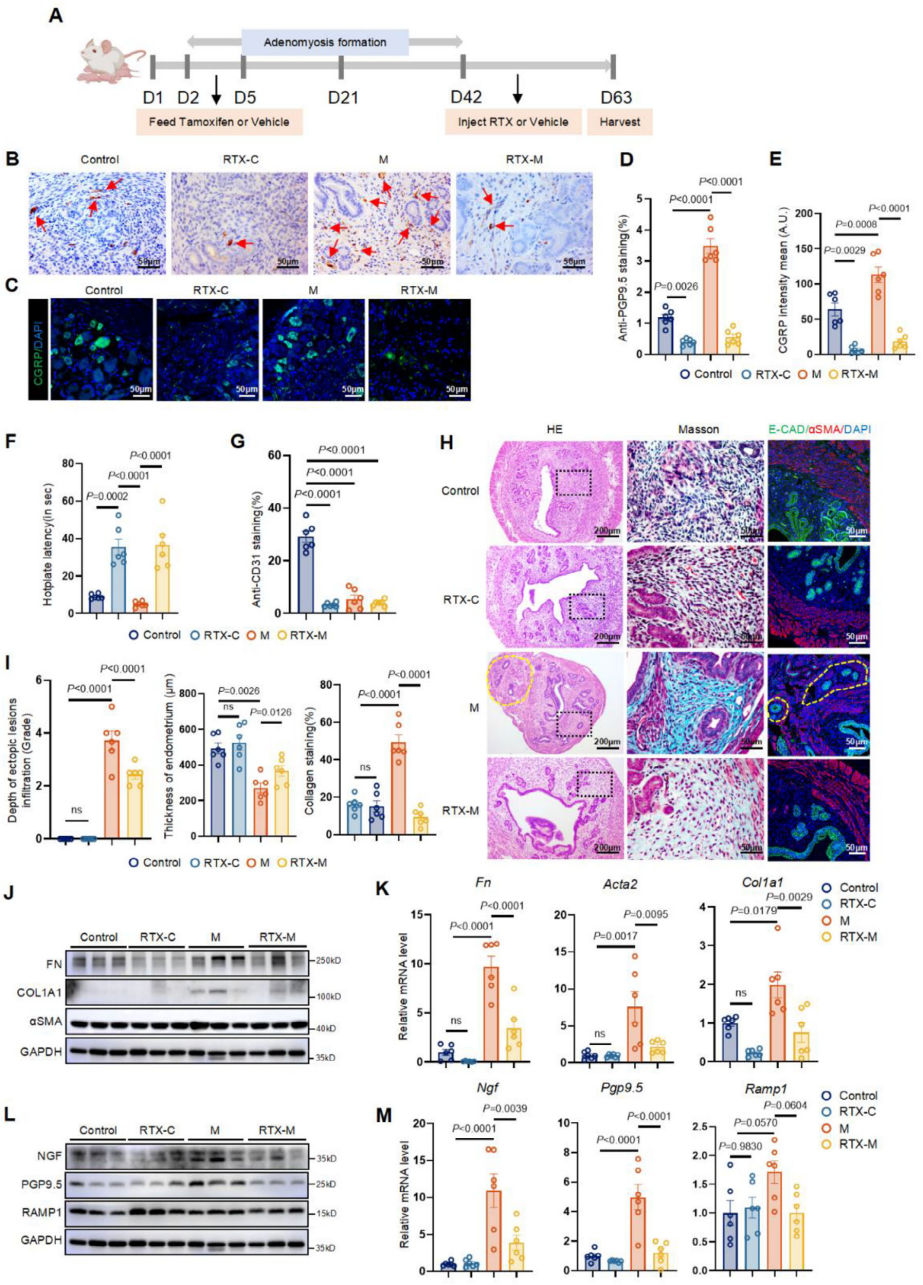
Nociceptive nerve ablation reduced uterine fibrosis in mice with induced AM. A) Schematic graph of the RTX‐mediated ablation of sensory nerve in mice with induced AM. (B) Representative images of IHC staining of uteri from mice with PGP9.5. Scale bar, 50 µm. Red arrows: positive PGP9.5 staining. C) Representative images of multiple IF staining of CGRP (green) and nucleus (DAPI, Blue) in DRG. Scale bar, 50 µm. D) Quantification of PGP9.5 staining. *n* = 6 per group. E) Quantification of CGRP intensity. *n* = 6 per group. F) Hotplate latency measured in Control, RTX‐C, M and RTX‐M groups. *n* = 6 per group. G) Quantification of CD31 staining. *n* = 6 per group. H) H&E, Masson trichrome staining of mice uteri at Day 63 and the corresponding horizon of multiple IF staining of E‐CAD (green), αSMA (red), and nucleus (DAPI, Blue). The yellow dotted circle showed the location of the ectopic lesion. The black dotted frame was the zoomed‐out version of the middle picture. Scale bar, left, 200 µm; middle, 50 µm; right, 50 µm. I) Score of the depth of ectopic lesion infiltration, eutopic endometrium, and semiquantitative analysis of the collagen areas of uterine tissue in Control, RTX‐C, M and RTX‐M groups. *n* = 6 per group. J) WB analysis of FN, COL1A1, and αSMA in Control, RTX‐C, M, and RTX‐M groups. *n* = 6 per group, three independent biological replicates on bands. K) Relative expression levels of *Fn*, *Col1a1*, *Acta2* (gene name of αSMA) in Control, RTX‐C, M and RTX‐M groups detected by qPCR. *n* = 6 per group. L) WB analysis of NGF, PGP9.5, and RAMP1 in Control, RTX‐C, M and RTX‐M groups. *n* = 6 per group, three independent biological replicates on bands. M) Relative expression levels of *Ngf*, *Pgp9.5*, and *Ramp1* in Control, RTX‐C, M and RTX‐M groups detected by qPCR. *n* = 6 per group. Results in D, E, F, G, I, K, M are expressed as the mean ± SEM, One‐way ANOVA. *p*‐value was shown in plots. RTX: resiniferatoxin; DRG: dorsal root ganglia; Control: control group of mice; RTX‐C: control mice receiving resiniferatoxin; M: mice with induced adenomyosis; RTX‐M: mice with induced adenomyosis receiving resiniferatoxin.

To further investigate the relationship between fibrosis and nociceptive nerves, we examined the effect of nociceptive nerve denervation in AM. A model of sensory nerve‐denervated mice was established using resiniferatoxin (RTX), as described in previous studies.^[^ ^19^, ^24^ ^]^ In total, 24 mice were included in this experiment and randomly allocated into four groups: Control, control mice receiving RTX (RTX‐C), mice with induced AM (M), and mice with induced AM receiving RTX (RTX‐M) (*n* = 6 per group). Uteri were harvested on Day 63 (**Figure** **2**A). IF analysis confirmed the absence of AM in both the Control and RTX‐C groups (Figure S3, Supporting Information).

Small nerve fibers were successfully ablated by RTX, as indicated by reduced expression of PGP9.5 and NGF in the uterus according to IHC and WB analysis (Figure 2B,D,L). IF analysis confirmed depletion of CGRP^+^ neurons in the DRG (Figure 2C,E). Functional assessment using hotplate tests revealed prolonged withdrawal latency of hind paws in RTX‐C and RTX‐M groups, suggesting impaired thermal sensation due to small nerve fiber ablation (Figure 2F).

The thermal sensitivity threshold was lower in the M group than in the Control group (Figure 2F). Histological sections of the uterus in the RTX‐M group showed a more regular myometrial layer, reduced ectopic lesion infiltration, increased endometrial thickness, and decreased fibrotic area compared with the M group (Figure 2H,I). Interestingly, blood vessel density was significantly decreased in both the RTX‐C and RTX‐M groups compared with the Control group (Figure 2G; Figure S6, Supporting Information).

Expression levels of FN, COL1A1, and αSMA, as well as NGF and PGP9.5, were decreased in the RTX‐M group compared with the M group, suggesting a role for nociceptive nerves in promoting fibrosis in AM (Figure 2J–M). RAMP1 expression did not significantly differ among the four groups (Figure 2L,M).

Collectively, these results demonstrated that ablation of CGRP^+^ nociceptive nerves ameliorated lesion development in mice with AM and indicated the involvement of nociceptive nerves in fibrotic progression in AM.

### 2.3 Single‐Cell Transcriptomic Analysis Revealed that CGRP Exerts Effects on Fibroblasts

**Figure 3:**
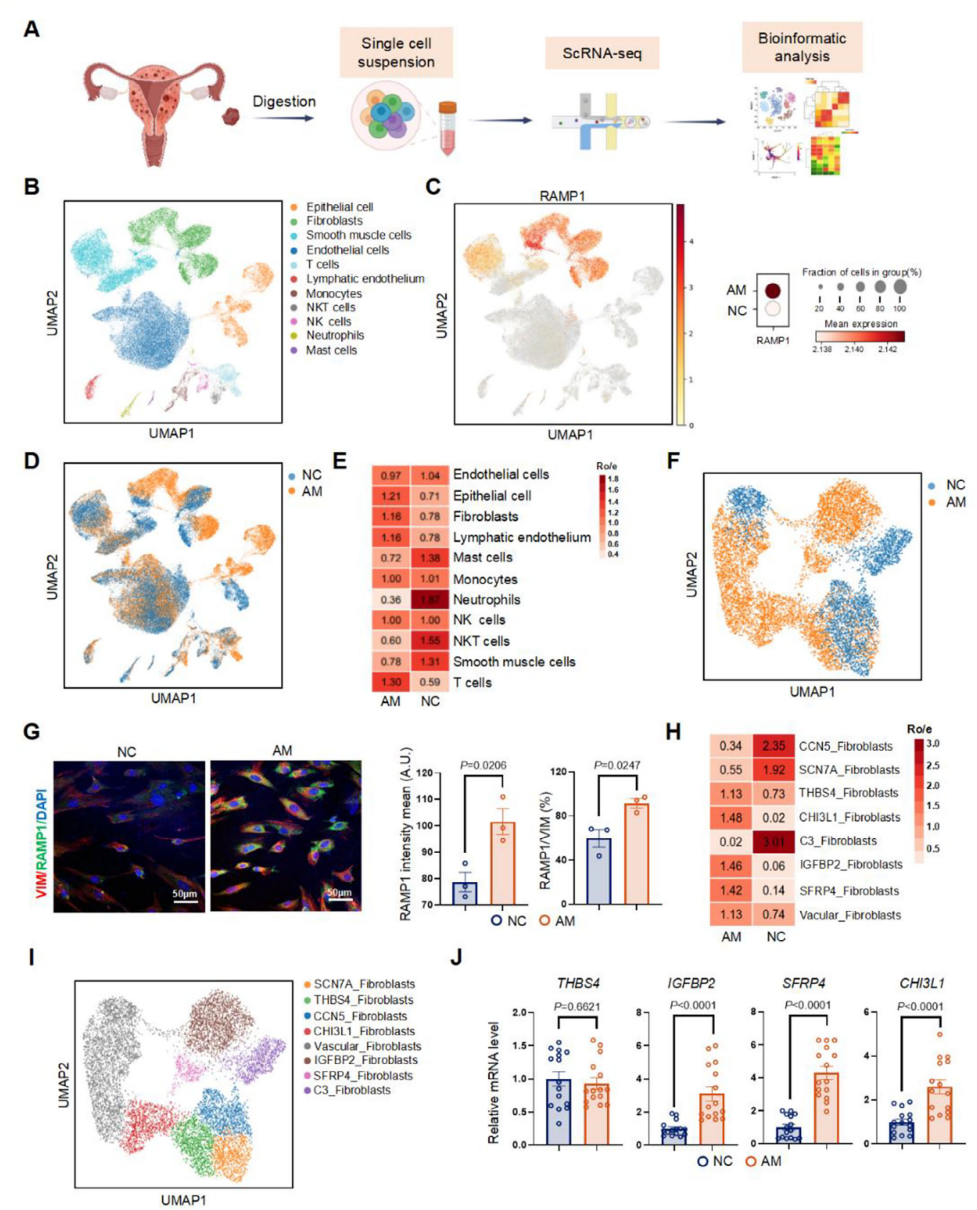
Single‐cell transcriptomic analysis revealed that CGRP exerts effects on fibroblasts. A) The workflow of the collection and processing of obtained myometrial samples from patients (NC, *n* = 3; AM, *n* = 3) for scRNA‐seq. Created by *BioRender*. B) Uniform manifold approximation and projection (UMAP) visualization of major cell types in AM and NC groups. C) The UMAP plot of RAMP1 distribution in all cell clusters and the dot plot of the relative expression of RAMP1 in AM and NC groups. D) The UMAP plot of the cell distribution of AM and NC. E) The Ro/e of 11 major cell types in AM and NC. F) The UMAP plot of the distribution of the fibroblast cluster in AM and NC. G) Representative images (left) and quantification (right) of multiple IF staining of Vimentin (VIM, a marker for fibroblasts, red), RAMP1 (green), and nucleus (DAPI, Blue) in the primary fibroblasts from NC and AM. Results are expressed as the mean ± SEM, *n* = 3 per group. Student's t‐test. *p*‐value was shown in plots. Scale bar, 50 µm. H) The Ro/e of each subgroup in fibroblasts of AM and NC. I) The UMAP plot of eight subclusters in fibroblasts. J) The relative mRNA expression levels of *THBS4*, *IGFBP2*, *SFRP4*, and *CHI3L1* in the AM and NC groups were detected by qPCR. Results are expressed as the mean ± SEM, *n* = 15 patients per group. Student's t‐test. *p*‐value was shown in plots. Ro/e: observed‐to‐expected cell ratios; NC: negative control group; AM: adenomyosis group.

Our results showed that CGRP^+^ nociceptive nerves contributed to fibrosis progression in AM. To further investigate the potential communication between CGRP and the ectopic lesion milieu, we performed scRNA‐seq on ectopic AM tissue (*n* = 3) from patients with progressive dysmenorrhea (Visual Analog Scale ≥4) and on healthy myometrial tissue from patients undergoing hysterectomy (NC, *n* = 3) (**Figure** **3**A). We analyzed the transcriptomic data of 53721 cells from these six patients. Cells with more than 200 expressed genes and a mitochondrial unique molecular identifier (UMI) rate below 20% passed quality filtering; mitochondrial genes were excluded from the expression table. In total, 48485 high‐quality cell profiles were obtained for downstream analysis; 5236 cells were excluded. The median number of detected genes was 2698. We constructed a single‐cell atlas by dimensionality reduction using uniform manifold approximation and projection (UMAP) and graph‐based clustering, which partitioned the cells into 26 clusters (Figure S7A, Supporting Information). Based on known lineage‐specific cell markers (Figure S7B, Supporting Information), the clusters were classified into 11 major cell types: fibroblasts, smooth muscle cells, endothelial cells, epithelial cells, lymphatic endothelial cells, monocytes, dendritic cells, T cells, NKT cells, mast cells, and neutrophils (Figure 3B; Figure S7C, Supporting Information). The top three differentially expressed genes for each cluster are displayed in a dot plot (Figure S7D, Supporting Information).

To identify cell types targeted by CGRP, we screened the clusters and observed that the fibroblast population was the primary RAMP1‐expressing subset. Compared with the NC group, RAMP1 expression was higher in the AM group (Figure 3C). Differences among 11 distinct cell types were noted between the NC and AM groups (Figure 3D). Analysis of observed‐to‐expected cell ratios for each cell type revealed that AM tissue contained a greater proportion of fibroblasts and a smaller proportion of smooth muscle cells, indicating altered cellular differentiation within the ectopic lesion milieu (Figure 3E). The fibroblast cluster was among the most noticeably different cell populations between the AM and NC groups (Figure 3F).

Multiple IF staining of primary cells verified that fibroblasts in the AM group expressed higher levels of RAMP1 (Figure 3G). Fibroblasts were classified into eight subgroups based on marker genes (Figure 3F,H,I; Figure S8, Supporting Information), using the classification framework for cancer‐associated fibroblasts.^[^ ^34^ ^]^ AM lesions contained greater proportions of *insulin‐like growth factor binding protein 2* (*IGFBP2)*‐, *secreted frizzled‐related protein 4* (*SFRP4*)‐, and *chitinase 3‐like 1* (*CHI3L1*)‐high‐expressing fibroblasts, consistent with qPCR verification (Figure 3H,J).

### 2.4 Single‐Cell Transcriptomic Analysis Revealed that a RAMP1^hi^ CD140b^+^ CD146^+^ Vascular_Fibroblast Subcluster Serves as the Effector Cell for CGRP and Differentiates into a CHI3L1_Fibroblast Subset in AM Lesions

**Figure 4:**
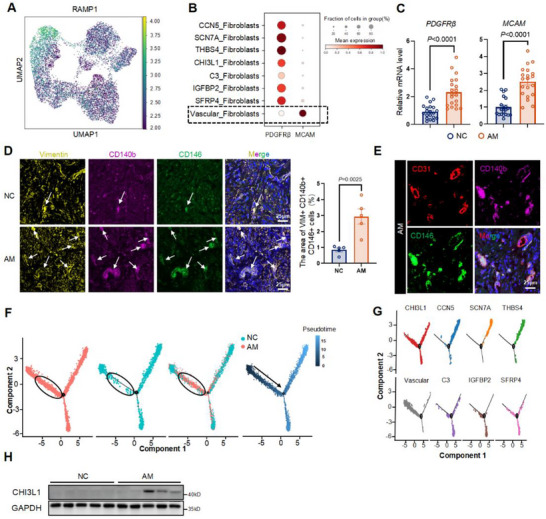
Single‐cell transcriptomic analysis revealed that a RAMP1^hi^ CD140b^+^ CD146^+^ Vascular_fibroblast subcluster serves as the effector cell for CGRP and differentiates into a CHI3L1_fibroblast subset in AM lesions. A) The UMAP plot of RAMP1 distribution in the fibroblast subcluster. B) The dot plot of *PDGFRβ* and *MCAM* gene expressions in different fibroblast subclusters. The dotted frame showed the subcluster that may simultaneously express *PDGFRβ* and *MCAM*. C) The relative expression levels of *PDGFRβ*, *MCAM* in AM and NC detected by qPCR. Results are expressed as the mean ± SEM, *n* = 20 patients per group. Student's t‐test. *p*‐values were shown in plots. D) Representative images (Left) and quantification (right) of multiple IF staining of Vimentin (VIM, a marker for fibroblasts, yellow), CD140b (gene name: *PDGFRβ*, violet), CD146 (gene name: *MCAM*, green), and nucleus (DAPI, Blue) in the myometrial tissue from AM and NC patients. *n* = 5 per group. Student's *t*‐test. *p*‐values were shown in plots. Scale bar, 25 µm. The white arrows show the cells coexpressing Vimentin, CD140b, and CD146. E) Representative images of multiple IF staining of CD140b (violet), CD146 (green), CD31 (red), and nucleus (DAPI, Blue) in the myometrium of AM. Scale bar, 25 µm. F) Trajectory of fibroblasts along pseudotime in a 2D space. The black circle denotes the cell group that is more abundant in the AM group. The black arrow denotes the direction of cell differentiation. G) The distribution of eight subclusters of fibroblasts in the pseudotime plot. H) WB detection of CHI3L1 from NC and AM patients. *n* = 5 independent biological replicates per group. NC: negative control group; AM: adenomyosis group.

Among the eight fibroblast subgroups, Vascular_Fibroblasts exhibited the highest RAMP1 expression density (Figures 3I and **4**A). Gene Ontology (GO) analysis of Vascular_Fibroblasts revealed enrichment in cell migration, axon guidance, angiogenesis, and myoblast differentiation (Figure S9, Supporting Information). Vascular_Fibroblasts also expressed high levels of melanoma cell adhesion molecule (*MCAM*; gene name for CD146), a marker of endometrial mesenchymal stem cells (eMSCs) (Figure 4B).^[^ ^52^, ^53^ ^]^ Another eMSC marker, platelet‐derived growth factor receptor beta (*PDGFRβ*; gene name for CD140b), was also detected in Vascular_Fibroblasts (outlined by a black dotted line in Figure 4B). However, *PDGFRβ* expression was not unique to the Vascular_Fibroblast cluster. To compare *PDGFRβ* expression differences between the two groups, we performed additional analysis of the single‐cell transcriptomic data. Notably, *PDGFRβ* expression was significantly higher in Vascular_Fibroblasts from the AM group than in those from the NC group (Figure S10, Supporting Information).

Furthermore, we performed a correlation analysis of *MCAM*, *PDGFRβ*, and *RAMP1* within the fibroblast cluster in AM and NC samples (Figure S11, Supporting Information). Positive correlations between *MCAM* and *PDGFRβ*, as well as between *MCAM* and *RAMP1*, were observed only in AM patients; no statistically significant correlations were detected in the NC group. These results indicated that Vascular_Fibroblasts expressing *RAMP1*, *MCAM*, and *PDGFRβ* might play a key role in CGRP‐mediated fibrosis in AM. In conjunction with the prevailing theories of endometrial invagination and stem cell differentiation in AM pathogenesis, we concluded that these specific Vascular_Fibroblasts in AM likely originate from the endometrium.

qPCR analysis of *PDGFRβ* and *MCAM* in 40 patient samples (NC, *n* = 20; AM, *n* = 20) confirmed that *PDGFRβ* and *MCAM* mRNA expression levels in AM lesions were increased compared with the NC group (Figure 4C). AM lesions also exhibited greater numbers of CD140b^+^ CD146^+^ fibroblasts coexpressing Vimentin, a fibroblast marker (Figure 4D; Figure S12, Supporting Information). To determine the location of this unique cell population within AM lesions, we performed multiple IF staining with CD31, a vascular marker, and found that CD140b^+^ CD146^+^ fibroblasts were adjacent to vessels (Figure 4E). This finding was consistent with the perivascular localization of CD140b^+^ CD146^+^ eMSCs in the endometrium.^[^ ^26^ ^]^

Thus, we focused on RAMP1^hi^ CD140b^+^ CD146^+^ Vascular_Fibroblasts in AM lesions. To further investigate the potential transitional processes of fibroblasts within the AM lesion milieu, we used Monocle2 to construct a pseudotime map of fibroblast developmental trajectories (Figure 4F). The Vascular_Fibroblast cluster was identified as the origin of the trajectories (Figure 4F,G). Differentiation trajectories differed between NC and AM groups. The CHI3L1_Fibroblast cluster was located downstream of Vascular_Fibroblasts (black circles, Figure 4F,G), while continuous differentiation from Vascular_Fibroblasts was not observed in the NC group.

CHI3L1 has demonstrated fibrosis and invasion involvement in various diseases^[^ ^35^, ^36^ ^]^ and may serve as a fibrotic biomarker.^[^ ^37^, ^38^ ^]^ Our GO analysis demonstrated that the CHI3L1_Fibroblast cluster was enriched in extracellular matrix organization, positive regulation of epithelial‐to‐mesenchymal transition, regulation of tissue remodeling, and regulation of response to wounding (Figure S13, Supporting Information). These biological processes were consistent with recurrent tissue damage and repair, as well as fibrosis observed in the AM lesion microenvironment. Ten myometrial samples from NC and AM patients (NC, *n* = 5; AM, *n* = 5) were evaluated by WB to determine CHI3L1 expression. The results showed higher CHI3L1 expression in the AM group (Figure 4H). Although CHI3L1 expression exhibited heterogeneity among AM patients, likely due to individual variation, it remained uniquely expressed in AM lesions and was absent in control tissues (Figures 3F,I and 4F–H; Figure S14, Supporting Information). Given that extracellular matrix deposition in the myometrium is a hallmark of AM, we concluded that CHI3L1_Fibroblasts represent a unique fibroblast subset in AM lesions that participates in the formation of the fibrotic milieu.

Taken together, we identified a unique population of RAMP1^hi^ CD146^+^ CD140b^+^ fibroblasts as effector cells of CGRP. These cells were activated and differentiated into a CHI3L1_Fibroblast subset, which ultimately promoted fibrosis in AM lesions.

### 2.5 CGRP Activates the Extracellular Signal‐Regulated Kinase (ERK) Signaling Pathway in RAMP1^hi^ CD140b^+^ CD146^+^ Fibroblasts and Promotes Extracellular Matrix Secretion

**Figure 5:**
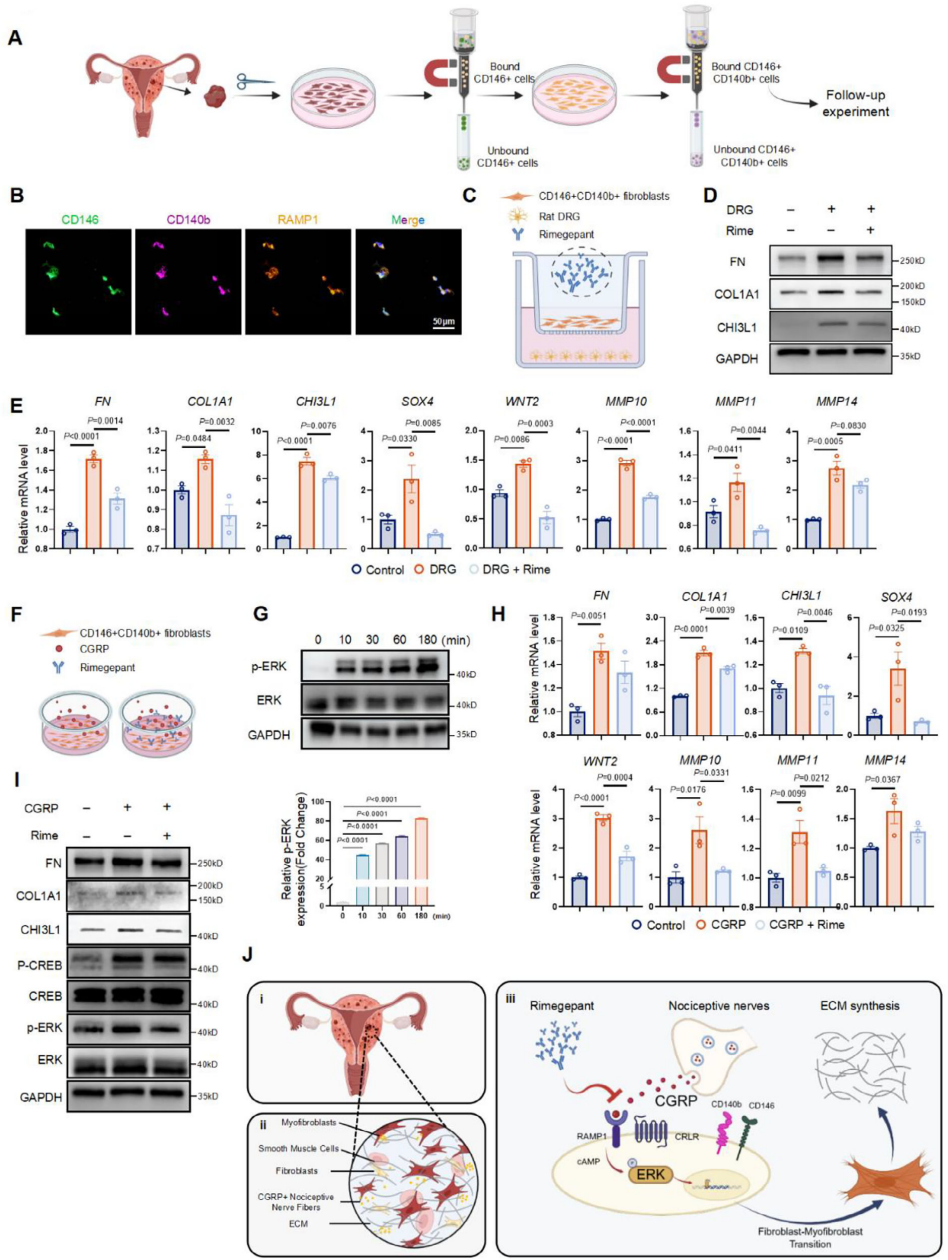
CGRP activates the extracellular signal‐regulated kinase (ERK) signaling pathway in RAMP1^hi^ CD140b^+^ CD146^+^ fibroblasts and promotes extracellular matrix secretion. A) Schematic illustration of the CD140b^+^ CD146^+^ fibroblasts isolation process. Created by *BioRender*. B) Representative images of multiple IF staining of CD140b (violet), CD146 (green), RAMP1 (orange), and nucleus (DAPI, Blue) in primary RAMP1^hi^ CD140b^+^ CD146^+^ fibroblasts. *n* = 3 independent biological replicates. Scale bar, 50 µm. C) Schematic illustration of DRG cocultured with RAMP1^hi^ CD140b^+^ CD146^+^ fibroblasts, with or without Rimegepant (Rime). Created by *BioRender*. D) Verify the effects of DRG on RAMP1^hi^ CD140b^+^ CD146^+^ fibroblasts. Representative images of WB detection of FN, COL1A1, and CHI3L1. *n* = 3 independent biological replicates. E) The relative expression levels of *FN*, *COL1A1*, *CHI3L1*, *SOX4*, *WNT2*, *MMP10*, *MMP11*, and *MMP14* were detected by qPCR. *n* = 3 per group. F) CGRP (100 nm, 24 h) was directly applied to RAMP1^hi^ CD140b^+^ CD146^+^ fibroblasts. Treatment with Rimegepant (100 nm) or vehicle was performed 30 min before the stimulus with CGRP. Created by *BioRender*. G) The relative expression levels (top) and quantification (bottom) of p‐ERK in a time‐dependent manner were detected by WB. *n* = 3 independent biological replicates per group. H) The relative expression levels of *FN*, *COL1A1*, *CHI3L1*, *SOX4*, *WNT2*, *MMP10*, *MMP11*, and *MMP14* were detected by qPCR. *n* = 3 per group. I) Representative images of effects and activated biological processes of CGRP on the RAMP1^hi^ CD140b^+^ CD146^+^ fibroblasts were assessed by WB. *n* = 3 independent biological replicates. J) Schematic diagram of how sensory nerve‐derived CGRP promotes RAMP1^hi^ CD140b^+^ CD146^+^ fibroblasts differentiation and participates in fibrosis in AM lesions. Created by *BioRender*. Results in E, G, and H are expressed as the mean ± SEM, One‐way ANOVA. *p*‐value was shown in plots.

To further investigate whether CGRP^+^ nociceptive nerves drive RAMP1^hi^ CD140b^+^ CD146^+^ fibroblasts to accelerate fibrosis progression, we extracted and cultured primary adenomyosis ectopic lesion‐derived fibroblasts (AMDFs) from three patients with AM. CD140b^+^ CD146^+^ fibroblasts were isolated from AMDFs using magnetic bead selection, as previously described (**Figure** **5**A).^[^ ^39^ ^]^ Magnetic selection efficiency was verified by flow cytometry (Figure S15, Supporting Information); coexpression patterns of CD140b, CD146, and RAMP1 markers were demonstrated by multiple IF staining (Figure 5B).

We established a coculture system in which DRG from rats were verified (Figure S16, Supporting Information) and cocultured with RAMP1^hi^ CD140b^+^ CD146^+^ fibroblasts, with or without the RAMP1 antagonist rimegepant, for 24 h (Figure 5C). In this DRG coculture system, protein and mRNA levels of FN, COL1A1, and CHI3L1 were increased in RAMP1^hi^ CD140b^+^ CD146^+^ fibroblasts (Figure 5D,E). In addition to characteristic CHI3L1 expression, CHI3L1_Fibroblasts exhibited high gene expression levels of *SRY‐box transcription factor 4* (*SOX4*), *Wnt family member 2* (*WNT2*), *matrix metalloproteinase 10* (*MMP10*), *MMP11*, and *MMP14* (Figure S17, Supporting Information). Previous studies have shown that the SOX and MMP families are associated with tissue remodeling, extracellular matrix homeostasis, cell invasion, and angiogenesis,^[^ ^40^, ^41^ ^]^ processes consistent with those observed in the AM lesional microenvironment. qPCR analysis confirmed upregulation of these genes after DRG coculture (Figure 5E). However, the addition of rimegepant reversed these effects (Figure 5D,E).

To further assess the molecular mechanism, we directly treated RAMP1^hi^ CD140b^+^ CD146^+^ fibroblasts with CGRP (100 nm, 24 h); rimegepant (100 nm) or vehicle was added 30 min before CGRP stimulation (Figure 5F). Phosphorylation of ERK was induced by CGRP treatment in a time‐dependent manner (Figure 5G). We also observed that fibrosis‐related protein and gene expression, along with phosphorylation involving both cyclic AMP response element‐binding protein (CREB) and ERK, were enhanced by CGRP treatment. These effects were inhibited by subsequent administration of rimegepant (Figure 5H,I). As an antagonist of the RAMP1 receptor, rimegepant also decreased expression of genes associated with CHI3L1_Fibroblasts, including *SOX4*, *WNT2*, *MMP10*, *MMP11*, and *MMP14* (Figure 5H).

Together, these results suggest that CGRP derived from nociceptive nerves facilitates the phenotypic switch of RAMP1^hi^ CD140b^+^ CD146^+^ fibroblasts into CHI3L1_Fibroblasts by activating the ERK pathway, thus accelerating lesion fibrosis in AM (Figure 5J).

### 2.6 Fibrosis Remission and Fertility Restoration were Induced by CGRP/RAMP1 Signaling Blockade in Mice

**Figure 6:**
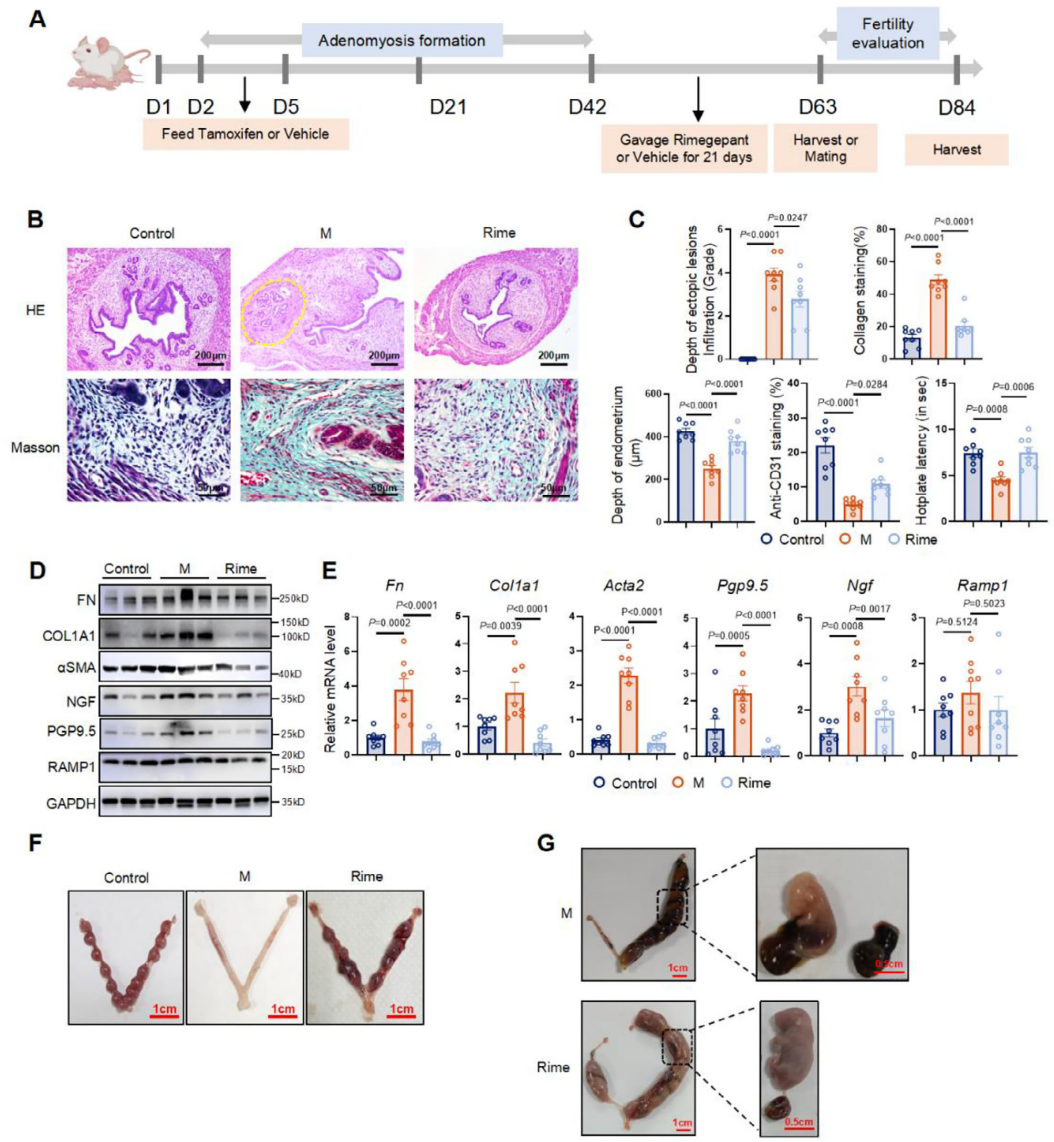
Fibrosis remission and fertility restoration were induced by CGRP/RAMP1 signaling blockade in mice. A) Schematic diagram of the in vivo study in the AM murine model. After 21 days of treatment with Rimegepant, the mice began to mate and fertility tests were conducted. During this period, Rimegepant or the vehicle was not administered. B) Representative images of mice uteri under different treatments at Day 63, examined by H&E staining and Masson trichrome staining. The yellow dotted line in the M group shows the location of the ectopic lesion. Scale bar, top, 200 µm; bottom, 50 µm. C) Measurement of the depth of the ectopic lesion infiltration, eutopic endometrium, semiquantitative analysis of the collagen areas by Masson trichrome staining, and anti‐CD31 staining by IHC in Control, M, and Rime groups. Hotplate latency measurement among three groups. Results are expressed as the mean ± SEM, *n* = 8 mice per group. One‐way ANOVA. *p*‐value was shown in plots. D) WB analysis of FN, COL1A1, αSMA, NGF, PGP9.5, RAMP1, GAPDH. *n* = 8 per group. Three independent biological replicates on bands. E) The relative expression levels of *Fn*, *Col1a1*, *Aata*2, *Ngf*, *Pgp9.5*, *Ramp1* in control, M and Rime groups were detected by qPCR. Results are expressed as the mean ± SEM, *n* = 8 mice per group. One‐way ANOVA. *p*‐value was shown in plots. F) Gross morphology of uterine horns from different groups in the fertility test. Scale bar, 1 cm. Control, *n* = 10; M, *n* = 27; Rime, *n* = 22. G) Representative mouse embryos from the M group and the Rime group at 18 embryonic days. Scale bar, left, 1 cm; right, 0.5 cm. Control: control group of mice; M: mice with induced adenomyosis; Rime: mice with induced adenomyosis and rimegepant treatment.

Based on preliminary evidence regarding CGRP^+^ nociceptive nerve effects on RAMP1^hi^ CD140b^+^ CD146^+^ fibroblasts, we conducted a rescue experiment in AM model mice. In total, 83 female mice were allocated into three groups: Control, mice with induced AM (M), and AM model mice treated with rimegepant (Rime). Mice were either sacrificed or included in fertility experiments after they had received rimegepant for 21 days (**Figure** **6**A). No signs of AM were observed in the Control group (Figure S3, Supporting Information).

Tissue samples were collected on Day 63. H&E staining revealed that ectopic lesion infiltration was ameliorated in the Rime group (*n* = 8) (Figure 6B,C). Masson trichrome staining was performed, and the collagen‐positive area was subjected to semi‐quantitative analysis. The fibrosis percentage in the Rime group was significantly lower (*p *< 0.0001) compared with the M (*n* = 8) group; it showed no significant difference relative to the Control (*n* = 8) group (Figure 6B,C). Endometrial thickness and blood vessel density were increased in the Rime group (Figure 6C; Figure S18, Supporting Information). Furthermore, the thermal pain threshold was restored in the Rime group, indicating pain relief (Figure 6C). Rimegepant significantly inhibited the expression of FN, COL1A1, αSMA, NGF, and PGP9.5 at both protein and gene levels compared with the M group (Figure 6D,E). RAMP1 expression showed no significant difference among the three groups (Figure 6D,E).

On Day 21 after mating, mice were sacrificed and their uteri were collected (Figure 6F). Notably, the Rime group (*n* = 22) demonstrated a higher pregnancy rate (50% versus 29.6%, *p* = 0.0395, Chi‐square test) and fewer miscarried embryos (15.8% versus 36.2%, *p* = 0.0048, Chi‐square test) compared with the M group (*n* = 27) (**Table** **1**; Table S1, Supporting Information). Observation of pregnant mice in late gestation revealed normal development in the Rime group, whereas the M group experienced miscarriage (Figure 6G). These findings indicated that rimegepant contributed to fertility restoration in AM model mice, possibly by maintaining extracellular matrix homeostasis via blockade of the CGRP‐RAMP1 axis.

**Table 1:** Reproductive outcomes after different treatments in a mouse model of adenomyosis.

Groups	Number of uteri	Number of pregnant uteri	Pregnant percentage (%)	Number of embryos	Number of miscarriages	Miscarriage percentage (%)
Control	20	14	70.0	85	5	5.6
M	54	16	29.6^a)^	44	25	36.2^b)^
Rime	44	24	50.0^c)^	64	12	15.8^d)^

^a)^ *p* = 0.0017, M vs Control, Chi‐square test;

^b)^ *p*< 0.0001, M vs Control, Chi‐square test;

^c)^ *p* = 0.0395, Rime vs M, Chi‐square test;

^d)^ *p* = 0.0048, Rime vs M, Chi‐square test. Control: control group of mice, *n* = 10; M: mice with induced adenomyosis, *n* = 27; Rime: mice with induced adenomyosis and rimegepant treatment, *n* = 22.

## 3 Discussion and Conclusion

AM is characterized by extensive fibrosis and progressive dysmenorrhea. The development and progression of AM involve multiple underlying pathophysiological mechanisms, including proliferation, migration, invasion, fibrosis, angiogenesis, inflammation, oxidative stress, immune response, and epigenetic alterations.^[^ ^42^ ^]^ Ectopic endometrial stromal cells secrete substantial quantities of NGF,^[^ ^43^ ^]^ which increases sensory nerve fiber density within lesions, thereby inducing excessive nerve regeneration and elevated expression of sensory neuropeptides. Several molecular mechanisms have been implicated in AM development, including ERK, WNT/β‐catenin, and phosphoinositide 3‐kinase/protein kinase B signaling pathways.^[^ ^3^ ^]^

In this study, we observed increased nociceptive nerve density; elevated CGRP, PGP9.5, and NGF expression levels; and enhanced fibrosis in the myometrium of AM samples from both patients and a mouse model. We also found that nociceptive nerve ablation in the mouse model ameliorated lesion infiltration and fibrosis. scRNA‐seq demonstrated that AM lesions contained fibroblasts with abnormal phenotypes. Within this population, the Vascular_Fibroblast cluster displayed the highest RAMP1 expression. Pseudotime analysis identified a group of unique cells within this cluster coexpressing CD140b and CD146 in AM lesions. Their perivascular localization, demonstrated by IF, was consistent with CD140b^+^ CD146^+^ eMSCs. Furthermore, in vitro experiments demonstrated that CGRP induces fibroblast‐to‐myofibroblast transition in RAMP1^hi^ CD140b^+^ CD146^+^ fibroblasts via the ERK signaling pathway. In the AM mouse model, rimegepant‐mediated blockade of the CGRP receptor RAMP1 alleviated fibrosis, reduced pain sensitivity, and increased the number of implanted embryos. Together, these results suggest that nociceptive nerve‐derived CGRP promotes fibrosis in AM by acting on RAMP1^hi^ CD146^+^ CD140b^+^ fibroblasts via the CGRP/RAMP1/ERK axis.

There is increasing evidence that nociceptive nerves and their secreted neuropeptides play key roles in chronic inflammation, fibrosis, and stem cell differentiation.^[^ ^44^, ^45^ ^]^ In endometriosis—a condition pathophysiologically analogous to AM—nociceptive nerves directly infiltrate lesions and accelerate fibrogenesis through neuropeptide signaling.^[^ ^19^, ^24^, ^46^ ^]^ Our findings are consistent with the published consensus, confirming involvement of nociceptive nerve‐derived CGRP in fibrosis within AM and highlighting nerve–fibroblast crosstalk as a critical amplifier of AM‐associated fibrogenesis.

As a chronic inflammatory disease, AM is associated with dysregulation of inflammatory signaling. Our sequencing results showed that AM lesions contained increased numbers of cytotoxic CD8^+^ T cells and abundant exhausted CD8^+^ T cells, consistent with recent findings.^[^ ^47^ ^]^ Although the number of cytotoxic CD8^+^ T cells increased in AM lesions, this was insufficient to prevent abnormal aggregation and persistence of ectopic endometrial cells in the myometrium. We consider this aggregation and persistence to be associated with the formation of an immunosuppressive microenvironment dominated by exhausted CD8^+^ T cells. CGRP, which functions as a neuropeptide in nociceptive nerves, also directly regulates immune cell differentiation,^[^ ^48^ ^]^ including the modulation of RAMP1‐expressing CD8^+^ T cell exhaustion, biasing differentiation toward Th1 cells, and polarizing macrophages toward a pro‐repair phenotype.^[^ ^49^, ^50^ ^]^ Thus, the crosstalk between CGRP^+^ nociceptive nerves and immune cells in AM lesions, along with its influence on disease progression, represents an important focus of future research.

ERK mediates the phosphorylation of transcription factors and regulates the expression of downstream target genes. Studies have shown that ERK/CREB or ERK/activator protein‐1 (AP‐1) signaling drives cell differentiation and fibroblast‐to‐myofibroblast transition in cancer and fibrotic diseases.^[^ ^51^, ^52^ ^]^ ERK‐mediated phosphorylation of transcription factors such as AP‐1 and CREB might allow their binding to the CHI3L1 promoter, thus regulating CHI3L1 transcription and expression. Additionally, our sequencing results and other studies have demonstrated that CHI3L1 activates ERK and the β‐catenin signaling pathway, contributing to cell survival, proliferation, epithelial–mesenchymal transition, and angiogenesis.^[^ ^35^ ^]^ CHI3L1 serves as a critical molecular nexus coordinating inflammation, injury, and repair responses; it is secreted by multiple cell types, including macrophages, epithelial cells, endothelial cells, fibroblasts, and stem cells.^[^ ^36^ ^]^ Our findings corroborate previous studies of CHI3L1 in endometriosis and AM.^[^ ^53^, ^54^ ^]^ We observed an increased CHI3L1_Fibroblasts proportion and elevated CHI3L1 expression in AM lesions, which contributed to extracellular matrix deposition and tissue remodeling. These results suggest that CHI3L1 is an important contributor to fibrosis in AM.

Notably, our scRNA‐seq analysis revealed distinct fibroblast subclusters within AM lesions. In addition to CHI3L1_Fibroblasts and Vascular_Fibroblasts, the AM group exhibited specific enrichment of SFRP4_Fibroblasts and IGFBP2_Fibroblasts compared with the NC group. This finding aligns with existing evidence implicating the IGFBP and SFRP families in AM pathogenesis.^[^ ^55^, ^56^ ^]^ The specific functional roles of these newly identified SFRP4^+^ and IGFBP2^+^ subpopulations, as well as their potential interactions with CHI3L1_Fibroblasts and Vascular_Fibroblasts, represent compelling avenues for future investigation to elucidate their collective contribution to AM progression.

We also observed increased nerve fiber density in the basal layer and junctional zone in the AM group compared with the NC group, consistent with previous findings.^[^ ^18^ ^]^ The role of hyperinnervated eutopic endometrium in stromal invasion and metastasis during the development of AM or endometriosis warrants further study.

This study had some limitations. First, because of individual variability in pain perception and disease progression, we did not assess degrees of dysmenorrhea or fibrosis. Second, nociceptive nerves also secrete substance P locally,^[^ ^57^ ^]^ which has been shown to accelerate lesion development in endometriosis.^[^ ^19^ ^]^ The effect of substance P on fibrosis in AM requires further investigation. Finally, we identified a unique fibroblast population in AM lesions expressing the same markers as eMSCs (CD140b^+^ and CD146^+^). Future studies should explore whether these cells possess stem cell properties.

In summary, we examined AM pathogenesis from the perspective of nociceptive nerves and disease progression. Using scRNA‐seq, we demonstrated that nociceptive nerves and their neuropeptide CGRP promote differentiation of RAMP1^hi^ CD140b^+^ CD146^+^ fibroblasts via the ERK pathway. Rimegepant, a CGRP/RAMP1 inhibitor, prevented lesion development and fibrosis, alleviated pain sensitivity, and preserved reproductive health and fertility in mice with AM. To our knowledge, this is the first study in AM to focus on nociceptive nerves and CD140b^+^ CD146^+^ fibroblasts. Our findings provide a novel perspective on the fibrotic pathomechanism of AM and highlight the potential of nonhormonal therapies targeting CGRP^+^ nociceptive nerves.

## 4 Experimental Section

### Animals

Animal studies were approved by the Animal Ethics Committee of Zhejiang University and the Ethics Committee of Sir Run Run Shaw Hospital, Zhejiang University School of Medicine (No. SRRSH202402295); they were conducted in accordance with the National Institutes of Health guidelines for animal care (NIH Publications No. 8023, revised 1978). Ten‐week‐old ICR‐Swiss pregnant mice, with a gestational age of 17–19 days, were obtained from the Animal Center of Sir Run Run Shaw Hospital and housed in a specific pathogen‐free environment at 23–25 °C with a 12/12 h light/dark cycle. During the remainder of gestation, each pregnant ICR mouse was housed in a single cage. Two days after birth, the pups were sexed, and female pups were selected for experiments. The mother and her litter were housed together until weaning, 3 weeks after birth.

### AM Model Establishment

AM was induced in mice as described by Parrott et al. and as previously reported.^[^ ^31^, ^58^ ^]^ Briefly, 2.5 mg tamoxifen (H31021545, Shanghai Fudan Forward Co. Ltd., China) was suspended in a peanut oil/lecithin/condensed milk mixture (2:0.2:3, by volume) to form a 0.2 mg mL^−1^ suspension. From Day 2 to Day 5 after birth, female neonatal mouse pups were separated from their mothers for 3 h daily to induce starvation and then drip‐fed tamoxifen at 1 mg kg^−1^ day^−1^ to establish the AM model. Randomly selected female control neonatal mice were treated identically but received only the solvent without tamoxifen. At 3 weeks of age, the female mice were weaned and separated from their mothers. Investigators conducting in vivo experiments were blinded to group allocation.

### Sensory Nerve Denervation

RTX (HY‐N2333, MedChemExpress, USA) was used to selectively ablate small, unmyelinated, predominantly C‐fiber sensory nerves. RTX was suspended in 10% Tween‐80 and 10% ethanol in sterile saline. Twenty‐four female mice were randomly assigned to four groups (*n* = 6 per group): 1) Control (vehicle + vehicle), 2) RTX‐C (vehicle + RTX), 3) M (tamoxifen + vehicle), and 4) RTX‐M (tamoxifen + RTX). Sensory denervation was induced by intraperitoneal injection of RTX at 150 µg/kg every 5 days, beginning on day 42. All mice were euthanized on day 63.

### Rescue Experiment

Eighty‐three female mice were allocated into three groups: 1) Control (*n* = 18; eight mice for histological and biochemical verification, 10 mice for fertility assessment, details in *Fertility assessment*); 2) induced AM (M, *n* = 35; eight mice for histological and biochemical verification, 27 mice for fertility assessment); and 3) rimegepant‐treated (Rime, *n* = 30; eight mice for histological and biochemical verification, 22 mice for fertility assessment). Rimegepant (T4610, TopScience, China), a CGRP receptor antagonist, was suspended in 0.5% sodium carboxymethylcellulose and administered orally (10 mg kg^−1^ day^−1^) for 3 weeks before euthanasia. The dose was calculated according to the metrological conversion formula between humans and mice and based on previous studies.^[^ ^59^ ^]^ The Control and M groups received vehicle gavage.

### Hotplate Test

To evaluate pain severity in mice, hotplate latency was used as a proxy for AM‐related hyperalgesia, consistent with previous endometriosis models.^[^ ^19^ ^]^ The hotplate test was performed with a commercial instrument (ZH‐YLS‐6BS, Anhui Zhenghua Biological Instrument Equipment Co. Ltd., China) as previously reported.^[^ ^19^ ^]^ Researchers were blinded to group allocation and given only animal identification codes.

Mice were acclimatized in the testing room for 1 h before testing. Hind paw withdrawal latency to thermal stimulation was determined. Each mouse was placed in the cylinder; latency (in s) was measured as the time between stimulus presentation and the first response, defined as lifting, licking, or withdrawing the paw. Latency was calculated as the mean of two sessions separated by 60 min. An automatic cutoff of 60 s was applied to avoid tissue damage.

### Fertility Assessment

Fifty‐nine female mice were assigned to three groups: Control (*n* = 10), induced AM (M, *n* = 27), and induced AM + rimegepant treatment (Rime, *n* = 22). After 21 days of rimegepant or vehicle gavage (day 63 after birth), mice were co‐housed with proven fertile males at a 2:1 ratio. The initial day of mating was designated gestational day 0. After 21 days of mating, uteri were exposed under anesthesia to confirm the presence of embryos. All mice were included in the fertility analysis.

### Patient Samples

This study was approved by the Ethics Committee of Sir Run Run Shaw Hospital, Zhejiang University School of Medicine (No. 20240215–231). All participants were recruited from the Department of Obstetrics and Gynecology, Sir Run Run Shaw Hospital, Zhejiang University School of Medicine, between March 2023 and May 2024. Written informed consent was obtained from all participants prior to enrollment. The study included 23 patients exhibiting AM accompanied by dysmenorrhea without endometriosis or uterine myoma, and 23 patients with uterine myoma or cervical carcinoma (NC group). All patients were premenopausal and had not received hormone therapy or intrauterine device placement for at least 6 months before surgery. Ectopic lesion samples were obtained from patients who underwent excision of AM lesions or hysterectomy; normal myometrial tissue was obtained from patients who underwent hysterectomy. Each patient's diagnosis was confirmed by pathological examination. The participants’ clinical characteristics are presented in Supplementary Table S2.

### ELISA

CGRP levels in myometrial tissue from patients (NC, *n* = 5; AM, *n* = 5) were quantified using a commercial ELISA kit (orb775074, Biorbyt, UK), in accordance with the manufacturer's instructions.

### Single‐Cell Suspension Preparation

Ectopic lesions were collected from three patients with AM and progressive dysmenorrhea (Visual Analog Scale ≥4); normal myometrial tissues were collected from three patients undergoing hysterectomy (details in Table S2, Supporting Information). Samples were washed twice with 1× Dulbecco's phosphate‐buffered saline (PBS) to remove residual tissue preservation solution; cut into 1‐mm^3^ pieces; and digested into single‐cell suspensions with Liberase TH (0.03 mg mL^−1^, 5401151001, Roche, Switzerland), collagenase type IV (1 mg mL^−1^, A004186, Sangon Biotech, China), dispase (0.03 mg mL^−1^, A002100, Sangon Biotech, China), and DNAase I (0.1 U µL^−1^, B006331, Sangon Biotech, China) in Roswell Park Memorial Institute (RPMI) 1640 (11835030, Gibco, USA) for 40 min at 200 rpm and 37 °C. The digested tissue was filtered through a 30‐µm cell strainer; cells were collected and washed three times at 300 g and 4 °C for 5 min. Supernatants were removed, and 1 mL of PBS containing 2.5% fetal bovine serum was added to resuspend the cells. A 10‐µL aliquot of the suspension was mixed with 10 µL of acridine orange/propidium iodide dye for cell counting using a Counterstar Rigel S2 analyzer (PerkinElmer Inc., USA). The suspension was treated with red blood cell lysis buffer at a ratio of 1:3 at 4 °C for 5 min. An equal volume of PBS was added to stop lysis; samples were centrifuged at 300 g and 4 °C for 5 min. Acridine orange/propidium iodide staining was repeated for cell counting. Cell viabilities in samples used for single‐cell RNA sequencing were 97%, 87%, and 90% for the AM group and 83%, 88%, and 89% for the NC group. The single‐cell suspensions were adjusted to 700–1200 cells µL^−1^.

### scRNA‐Seq

Single‐cell suspensions were loaded onto a Chromium Single‐Cell Controller Instrument (10× Genomics) to generate single‐cell gel beads in emulsions. Reverse transcription and library construction were performed in accordance with the manufacturer's instructions. Reverse transcription generated barcoded full‐length cDNA, which was recovered and enriched by PCR amplification for cDNA library construction. Final libraries were sequenced on a NovaSeq 6000 platform (Illumina, USA).

Cell Ranger (version 7.1.0; 10× Genomics) was used to process raw data, demultiplex cellular barcodes, map reads to the transcriptome, and downsample reads, as required, for the generation of normalized aggregate data across samples. The Scanpy package (version 1.9.3, https://scanpy.readthedocs.io/en/stable/) was used for cell normalization and regression based on the expression table, according to UMI counts of each sample and mitochondrial gene percentage, yielding scaled data. Principal component analysis was performed on the scaled data using the top 2000 highly variable genes; the top 10 principal components were used for tSNE and UMAP construction. Unsupervised cell clustering was performed using a graph‐based method with the top 30 principal components from principal component analysis. Marker genes were calculated using the scanpy.tl.rank_genes_groups function with the Wilcoxon rank‐sum test algorithm under the following criteria: log_2_(fold change) >1, *p* < 0.05, and pct_nz_group >0.25. To refine cell type identification, clusters of the same cell type were subjected to repeat UMAP analysis, graph‐based clustering, and marker analysis.

GO analysis was performed to elucidate the biological implications of marker genes and differentially expressed genes.^[^ ^60^ ^]^ GO annotations were downloaded from the National Center for Biotechnology Information (http://www.ncbi.nlm.nih.gov/), UniProt (http://www.uniprot.org/), and GO (http://www.geneontology.org/). Fisher's exact test was utilized to identify significant GO categories, and false discovery rate correction was applied to *p*‐values.

Pseudotime analysis was performed in an unsupervised manner. Monocle2 (http://cole‐trapnell‐lab.github.io/monoclerelease) was used to conduct single‐cell trajectory analysis; DDR‐Tree and default parameters were implemented. Prior to Monocle analysis, marker genes were selected from Seurat clustering results, and raw expression counts of filtered cells were used. Branch expression analysis modeling was conducted to identify branch fate‐determining genes, based on the results of the pseudotime analysis.

### Isolation and Culture of Primary Adenomyosis Ectopic Lesion‐Derived Fibroblasts (AMDFs)

Ectopic lesion tissue was obtained from three women with AM (details in Table S2, Supporting Information) for cell extraction and culture. In accordance with previously published methods,^[^ ^61^ ^]^ samples were washed twice with PBS, cut into <1‐mm^3^ pieces, and incubated in Dulbecco's modified Eagle medium (DMEM)/F12 supplemented with collagenase IV (4 mg mL^−1^, 17104019, Gibco, USA) and DNase I (0.01 mg mL^−1^, 11284932001, Sigma–Aldrich, USA) in a 37 °C incubator with shaking for 60 min. The reaction was terminated by adding an equal volume of DMEM/F12 complete medium (DMEM/F12 with 10% fetal bovine serum [FBS; SA102.02, CellMax, China]). Samples were diluted with PBS to twice the original volume, and each suspension was successively filtered through 100‐µm (BS‐100‐CS, Biosharp, China) and 40‐µm mesh filters (BS‐40‐CS, Biosharp, China) to obtain isolated fibroblasts. The filtrate was centrifuged at 1200 rpm for 8 min, the supernatant was removed, and the cell pellet was resuspended in DMEM/F12 complete medium. Cells were cultured at 37 °C in a humidified incubator with 5% CO_2_. When cells became adherent, the medium was replaced every 2 days.

### Magnetic Selection of CD146^+^ CD140b^+^ Cells and Flow Cytometry Confirmation

Adenomyosis ectopic lesion‐derived fibroblasts (AMDFs) at 80% confluence were used for sorting of CD140b^+^ CD146^+^ cells, in accordance with previously described methods.^[^ ^39^ ^]^ After primary fibroblast expansion in vitro, fibroblasts were incubated with anti‐CD146 antibody‐coated microbeads (130‐093‐596, Miltenyi Biotec, Germany) for 15 min at 4 °C. The CD146^+^ population was collected by applying cell suspensions to MS columns (130‐042‐201, Miltenyi Biotec, Germany) in a magnetic field. CD146^+^ fibroblasts were then cultured in fibronectin (33016015, Gibco, USA)‐coated dishes with DMEM/F12 complete medium at 37 °C in 5% CO_2_. When cells reached 80% confluence, they were trypsinized and incubated with phycoerythrin (PE)‐conjugated anti‐CD140b antibody (FAB1263P, R&D Systems, USA) for 45 min at 4 °C, then incubated with anti‐mouse IgG1‐coated magnetic microbeads (130‐047‐102, Miltenyi Biotec, Germany) for 15 min at 4 °C. CD140b^+^ CD146^+^ fibroblasts were subsequently retained in the column and flushed out for use. A subset of the cells was stained with fluorescein isothiocyanate‐conjugated anti‐CD146 (361011, BioLegend, USA) and PE‐conjugated anti‐CD140b (323605, BioLegend, USA) antibodies to assess selection efficiency by flow cytometry. The remaining fibroblasts were used for further experiments at passages 1–4.

### Isolation and Culture of DRG

DRG cells were isolated as previously described.^[^ ^62^ ^]^ Briefly, three 3‐week‐old female Sprague–Dawley rats were euthanized, and DRG from the L2–L5 spinal levels were isolated in cold DMEM (11995065, Gibco, USA) containing 100 U mL^−1^ penicillin, 100 µg mL^−1^ streptomycin (23355699, Biosharp, China), and 10% FBS. Tissues were treated with 1 mg mL^−1^ collagenase type IV and 2.4 U mL^−1^ dispase II (04942078001, Sigma–Aldrich, USA) at 37 °C for 80 min. Ganglia were successively triturated using 22G, 26G, and 29G syringe needles; the suspension was centrifuged over a 15% bovine serum albumin gradient. Cells collected at the bottom of the tube were plated on poly‐l‐lysine (P2100, Solarbio, China) –coated six‐well plates in DMEM with 10% FBS. Six hours later, the medium was replaced with Neurobasal‐A medium (21103049, Gibco, USA) supplemented with 0.05 ng µL^−1^ NGF (13257‐019, Gibco, USA), 0.002 ng µL^−1^ glial cell line‐derived neurotrophic factor (450‐51‐2, PeproTech, USA), 200 mm L‐glutamine (A2916801, Gibco, USA), and B‐27 supplement (17504044, Gibco, USA). On day 3, 0.01 mm cytosine arabinoside (C6645, Sigma–Aldrich, USA) was added to the Neurobasal culture medium to decrease glial cell proliferation. Half of the culture medium was replaced every 3 days to gradually lower the cytosine arabinoside concentration and maintain neuronal growth.

### Reverse Transcription and qPCR

Total RNA was purified from cells or tissues using TRIzol (R401‐01, Vazyme, China), in accordance with the manufacturer's instructions. RNA (1 µg) was reverse transcribed with HiScript II Q RT SuperMix for qPCR (R222‐01, Vazyme, China), and qPCR was performed using 2× ChamQ Universal SYBR qPCR Master Mix (Q711‐02, Vazyme, China) on the CFX96 and CFX384 systems (Bio‐Rad, USA). Primer sequences are listed in Supplementary Table S3. Fold change in expression relative to the housekeeping gene was calculated using the 2^−ΔΔCT^ method.

### WB Analysis

Protein samples were harvested from tissues and cells using ice‐cold radioimmunoprecipitation assay (RIPA) lysis buffer (R0010, Solarbio, China) supplemented with 1% phenylmethanesulfonyl fluoride (ST506, Beyotime, China), protein phosphatase inhibitor (P1260, Solarbio, China), and protease inhibitor mixture (PP6730, Solarbio, China). Protein concentrations were determined using the Pierce BCA Protein Assay Kit (23225, Thermo Fisher, USA), in accordance with the manufacturer's instructions. Concentrations were calculated by interpolation from a standard curve and adjusted to 6 µg µL^−1^ with RIPA buffer. Protein samples (30 µg per lane) were separated by sodium dodecyl sulfate‐polyacrylamide gel electrophoresis and transferred to polyvinylidene fluoride membranes (ISEQ00010, Millipore, USA). Membranes were blocked with 5% skim milk (232100, BD Difco, USA) at room temperature for at least 1 h, then incubated with primary antibodies overnight at 4 °C. Primary antibodies in this study recognized fibronectin (F3648, 1:1000, Sigma–Aldrich, USA), COL1A1 (66948, 1:1000, Cell Signaling Technology, USA), αSMA (19 245, 1:1000, Cell Signaling Technology, USA), NGF (ab52918, 1:1000, Abcam, USA), CGRP (ab283568, 1:1000, Abcam, USA), RAMP1 (ab156575, 1:1000, Abcam, USA), PGP9.5 (ab108986, 1:1000, Abcam, USA), CHI3L1 (12036‐1‐AP, 1:1000, Proteintech, China), phosphorylated ERK (4370, 1:1000, Cell Signaling Technology, USA), ERK (4695, 1:1000, Cell Signaling Technology, USA), phosphorylated CREB (9198, 1:1000, Cell Signaling Technology, USA), CREB (9197, 1:1000, Cell Signaling Technology, USA), and glyceraldehyde‐3‐phosphate dehydrogenase (60004‐1‐Ig, 1:5000, Proteintech, China). After membranes had been washed in Tris‐buffered saline with Tween‐20, they were incubated with horseradish peroxidase (HRP)‐conjugated goat anti‐mouse secondary antibody (115‐035‐003, 1:5000, Jackson ImmunoResearch, USA) or HRP‐conjugated goat anti‐rabbit secondary antibody (111‐035‐003, 1:5000, Jackson ImmunoResearch). Protein bands were visualized by enhanced chemiluminescence (WBKLS0500, Millipore, USA), and images were captured with a ChemiDoc Touch Imaging System (Bio‐Rad, USA).

### IHC

Slides were dewaxed with xylene for 20 min and sequentially rehydrated in 100%, 95%, and 75% ethanol for 20 min. Antigen retrieval was performed in antigen retrieval buffer (citrate sodium buffer, pH 6.0, or Tris/ethylenediaminetetraacetic acid buffer, pH 9.0) at 100 °C for 10 min. Endogenous peroxidase activity was blocked with 3% H_2_O_2_; slides were then blocked with 5% bovine serum albumin for 1 h at room temperature. Slides were incubated overnight at 4 °C with primary antibodies against PGP9.5 (ab108986, 1:250, Abcam, USA), CGRP (ab283568, 1:500, Abcam, USA), and CD31 (ab28364, 1:50, Abcam, USA) in 5% bovine serum albumin; rinsed; and incubated with HRP‐conjugated secondary antibody for 30 min. Staining was developed with 3,3′‐diaminobenzidine tetrahydrochloride solution. Nuclei were counterstained with hematoxylin (C0107, Beyotime, China) for 5 min. Slides were examined under an optical microscope, and five high‐power fields were randomly selected for analysis.

### IF

Slides were dewaxed and rehydrated; antigen retrieval was performed as described above. Sections were permeabilized and blocked with 0.3% (v/v) Triton X‐100 in 5% (w/v) bovine serum albumin for 1 h. Samples were incubated overnight at 4 °C with primary antibodies against TUBB3 (657403, 1:100, BioLegend, USA), CGRP (ab283568, 1:100, Abcam, USA), E‐Cadherin (14472, 1:100, Cell Signaling Technology, USA), αSMA (19245, 1:500, Cell Signaling Technology, USA), PGP9.5 (ab108986, 1:250, Abcam, USA), Vimentin (MA5‐11883, 1:200, Invitrogen, USA), RAMP1 (ab156575, 1:100, Abcam, USA), PE‐conjugated anti‐human CD140b (323605, 1:100, BioLegend, USA), and fluorescein isothiocyanate‐conjugated anti‐human CD146 (361011, 1:100, BioLegend, USA). Slides were then incubated for 1 h with secondary antibodies, including Alexa Fluor 568 goat anti‐rabbit (A‐11036, 1:500, Thermo Fisher, USA), Alexa Fluor 568 goat anti‐mouse (A‐11031, 1:500, Thermo Fisher, USA), Alexa Fluor 488 goat anti‐mouse (A11029, 1:500, Thermo Fisher, USA), Alexa Fluor 488 goat anti‐rabbit (A11034, 1:500, Thermo Fisher, USA), Alexa Fluor 647 goat anti‐rabbit (A21245, 1:500, Thermo Fisher, USA), and Alexa Fluor 647 goat anti‐mouse IgG (A21236, 1:500, Thermo Fisher, USA). Nuclei were stained with Hoechst 33342 (H3570, 1:1000, Invitrogen, USA) for 15 min. Samples were imaged by laser scanning confocal microscopy (LSM800, Zeiss, Germany). For tissue samples, the TrueVIEW Autofluorescence Quenching Kit (SP‐8400, Vector Laboratories, USA) was utilized before nuclear staining to eliminate autofluorescence caused by red blood cells and collagen fibers.

### Histological Analysis

The estrous cycle stage was determined before euthanasia by routine vaginal smear cytology. Only mice confirmed to be in the proliferative phase were included in analyses of endometrial thickness and other uterine parameters. Samples collected from mice and patients were washed twice with Dulbecco's PBS and fixed in 4% paraformaldehyde. Tissues were dehydrated, embedded in paraffin, sectioned at 5 µm thickness, and stained with H&E or Masson trichrome. Sections were examined under an optical microscope to evaluate tissue infiltration and fibrosis. Progression of AM in mice was classified in accordance with the method used by Mori et al.:^[^ ^63^ ^]^ grade 0, normal uterus; grade 1, invasion of endometrial stromal cells into the inner myometrium; grade 2, invasion of endometrial stromal and gland cells into the inner myometrium; grade 3, invasion of endometrial stromal and gland cells into the connective tissue space between the inner and outer myometrial layers; grade 4, cystic hyperplasia of endometrial glands with small nodules beneath the serosa; and grade 5, cystic hyperplasia of endometrial glands with numerous subserosal nodules. At least five images from each mouse were captured and analyzed. All images were evaluated independently by two observers. Endometrial thickness was measured using ImageJ software. A straight line was drawn from the center of the uterine cavity to the myometrium (for AM‐induced mice, the endpoint was defined as the myometrium adjacent to the endometrium, excluding ectopic lesions). The length of this line was recorded as one measurement of endometrial thickness. For each slide, measurements were taken at eight different angles; the average value was calculated as the endometrial thickness for that slide.

### Statistical Analysis

Data are expressed as mean ± standard error of the mean (SEM). Statistical analyses between the two groups were performed using two‐sided Student's *t*‐tests. For experiments involving more than two groups, analysis of variance (ANOVA) followed by Tukey's post hoc test was conducted. Categorical variables, expressed as absolute numbers or percentages, were assessed using the Chi‐square test. All statistical analyses were performed with GraphPad Prism 8.0 software (GraphPad Software, Inc., USA). *p*‐values less than 0.05 were considered statistically significant, and specific *p*‐values are indicated in the figures. ImageJ software was used to quantify image intensities; at least four high‐power fields per slide were analyzed. Statistical analyses and sample sizes for each experiment are provided in the figure legends.

## Conflict of Interest

The authors declare no competing interests.

## Author Contributions

L.M., S.Z., and L.X. performed the conceptualization. Z.Y., L.M., S.Z., and L.X. performed the investigation. Z.Y. and A.Z. developed the methodology. L.X. administered the project. L.M. and S.Z. supervised the study. Z.Y., A.Z., Y.D., T.L., and W.S. visualized the study. X.L., Y.H., D.H., J.C., and L.X. acquired the human sample. Z.Y. wrote the original draft. All the authors wrote, reviewed, and edited the final draft. S.Z. and L.X. acquired funds. All authors read and approved the final version of the manuscript.

## Supporting information



Supporting Information

## Data Availability

The data that support the findings of this study are available from the corresponding author upon reasonable request. The raw sequence data reported in this paper have been deposited in the Genome Sequence Archive (Genomics, Proteomics & Bioinformatics 2025) in National Genomics Data Center (Nucleic Acids Res 2025), China National Center for Bioinformation/Beijing Institute of Genomics, Chinese Academy of Sciences (GSA‐Human: PRJCA045935) that are publicly accessible at https://ngdc.cncb.ac.cn/gsa‐human.
