# Supplementary material for: Nociceptive Nerve‐Derived CGRP Exacerbates Uterine Fibrogenesis in Adenomyosis by Promoting CD140b+ CD146+ Fibroblast Differentiation
Source: Adv Sci (Weinh). 2025 Sep 23;12(45):e07128. doi: 10.1002/advs.202507128 (PMC12677688; doi:10.1002/advs.202507128)
Supplement: Supplementary file 1 — Supporting Information [file ADVS-12-e07128-s001.pdf]

Supporting Information

Nociceptive nerve-derived CGRP exacerbates uterine fibrogenesis in adenomyosis by promoting CD140b$^+$ CD146$^+$ fibroblasts differentiation

Zi Ye, Anning Zhao, Xia Li, Yanqing Hao, Dong Huang, Jianmin Chen, Tiantian Li, Yangyang Dai, Wenchao Sun, Lie Ma, Songying Zhang*, Liaobing Xin**

Affiliation

Zi Ye, Anning Zhao, Xia Li, Yanqing Hao, Dong Huang, Jianmin Chen, Tiantian Li, Yangyang Dai, Songying Zhang, Liaobing Xin

Assisted Reproduction Unit, Department of Obstetrics and Gynecology, Sir Run Run Shaw Hospital, School of Medicine, Zhejiang University, Hangzhou, 310016, China.

Zhejiang Provincial Clinical Research Center for Reproductive Health and Disease, Hangzhou, 310016, China.

Zi Ye, Anning Zhao, Xia Li, Yanqing Hao, Dong Huang, Jianmin Chen, Tiantian Li, Yangyang Dai, Lie Ma, Songying Zhang, Liaobing Xin

Zhejiang Key Laboratory of Precise Protection and Promotion of Fertility, Hangzhou, 310016, China.

Zi Ye, Lie Ma

MOE Key Laboratory of Macromolecular Synthesis and Functionalization, Department of Polymer Science and Engineering, Zhejiang University, Hangzhou, 310027, China.

Wenchao Sun

Department of Reproductive Endocrinology, Hangzhou Women's Hospital, Hangzhou, 310001, China.

*Co-corresponding author: Lie Ma, Songying Zhang, Liaobing Xin

E-mail: liema@zju.edu.cn, zhangsongying@zju.edu.cn, 11618241@zju.edu.cn.

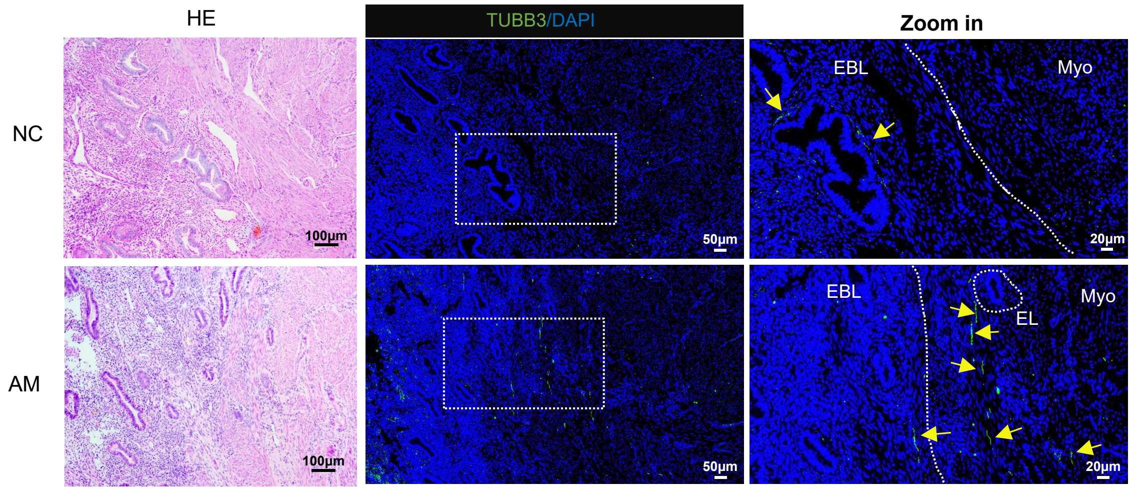

Supplementary Figure S1. Representative images of IF of TUBB3 (Green) corresponding to HE images in full-thickness endometrium, without and with adenomyosis. EBL: Endometrium Basal Layer, Myo: Myometrium Layer, EL: Ectopic Lesions. NC: negative control group, AM: adenomyosis group. n=5 patients in each group. Arrows: nerve fibers. Scale bar, left, 100 µm; middle, 50µm; right, 20µm.

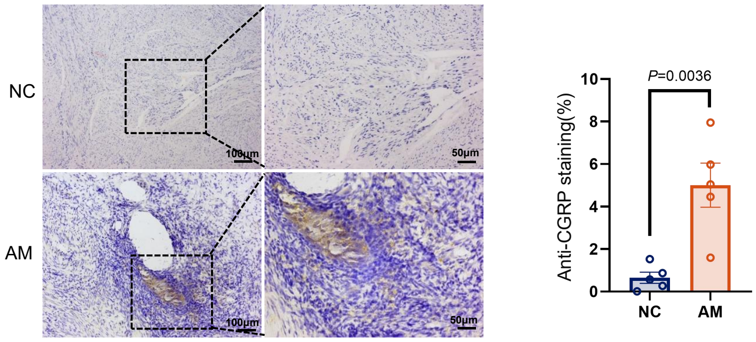

Supplementary Figure S2. Representative images and quantification of IHC staining with anti-CGRP staining in the myometrium in control and adenomyosis lesions from patients. n=5 per group. Data are represented as mean ± SEM, and analyzed by Student's t-test. *P*-value were shown in plots. Scale bar, left, 100 μm; right, 50 μm. NC: negative control group, AM: adenomyosis group.

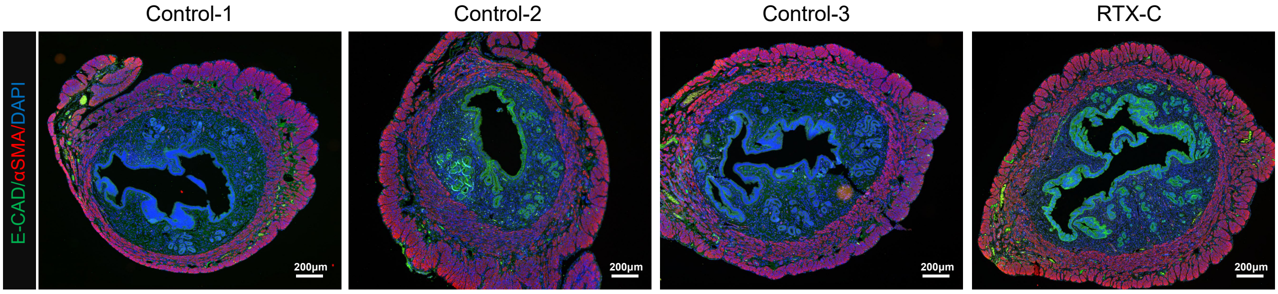

Supplementary Figure S3. Representative images of immunofluorescence of E-cadherin (E-CAD, marker of epithelial cells, Green), αSMA (marker of smooth muscle cells, red) in uterus from mice to confirm the absence of AM in Control and RTX-C groups. Scale bar, 200 μm. Control-1: the uteri of mice from the control group in Figure 1 (n=10); Control-2: the uteri of mice from the control group in Figure 2, (n=6); Control-3: the uteri of mice from the control group in Figure 6, (n=8); RTX-C: the uteri of mice from the control group receiving resiniferatoxin in Figure 2, (n=6).

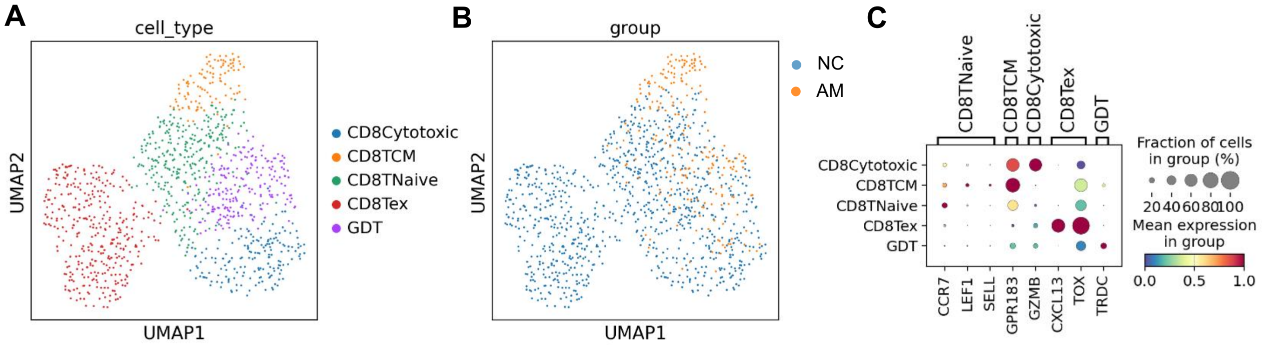

Supplementary Figure S4. A) The distribution of 5 subclusters of CD8+ T cells by UMAP plots. **B)** The UMAP plot of the distribution of CD8+ T cells in AM and NC. **C)** Expression of typical marker genes of each CD8+ T cell subcluster.

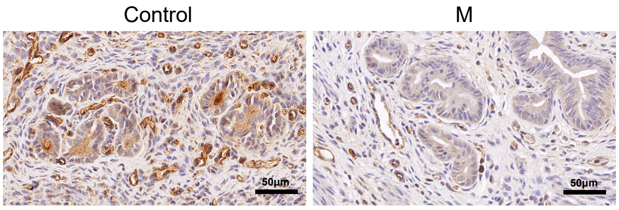

Supplementary Figure S5. Representative images of IHC staining of uteri from mice with CD31 in Control and M groups. n = 10 per group. Scale bar, 50μm. Control: control group of mice; M: mice with induced adenomyosis.

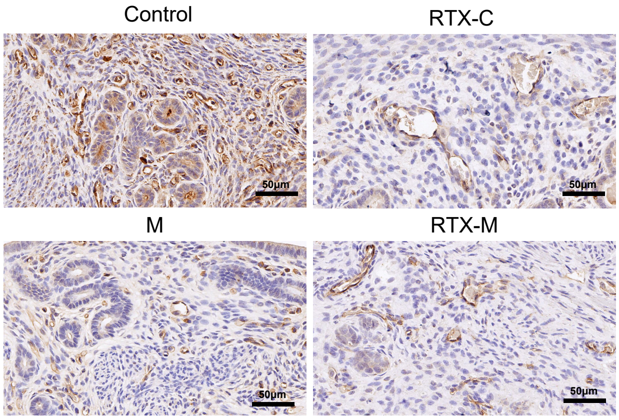

Supplementary Figure S6. Representative images of IHC staining of uteri from mice with CD31 in Control, RTX-C, M and RTX-M groups. n = 6 per group. Scale bar, 50μm. Control: control group of mice; RTX-C: control mice receiving resiniferatoxin; M: mice with induced adenomyosis; RTX-M: mice with induced adenomyosis receiving resiniferatoxin.

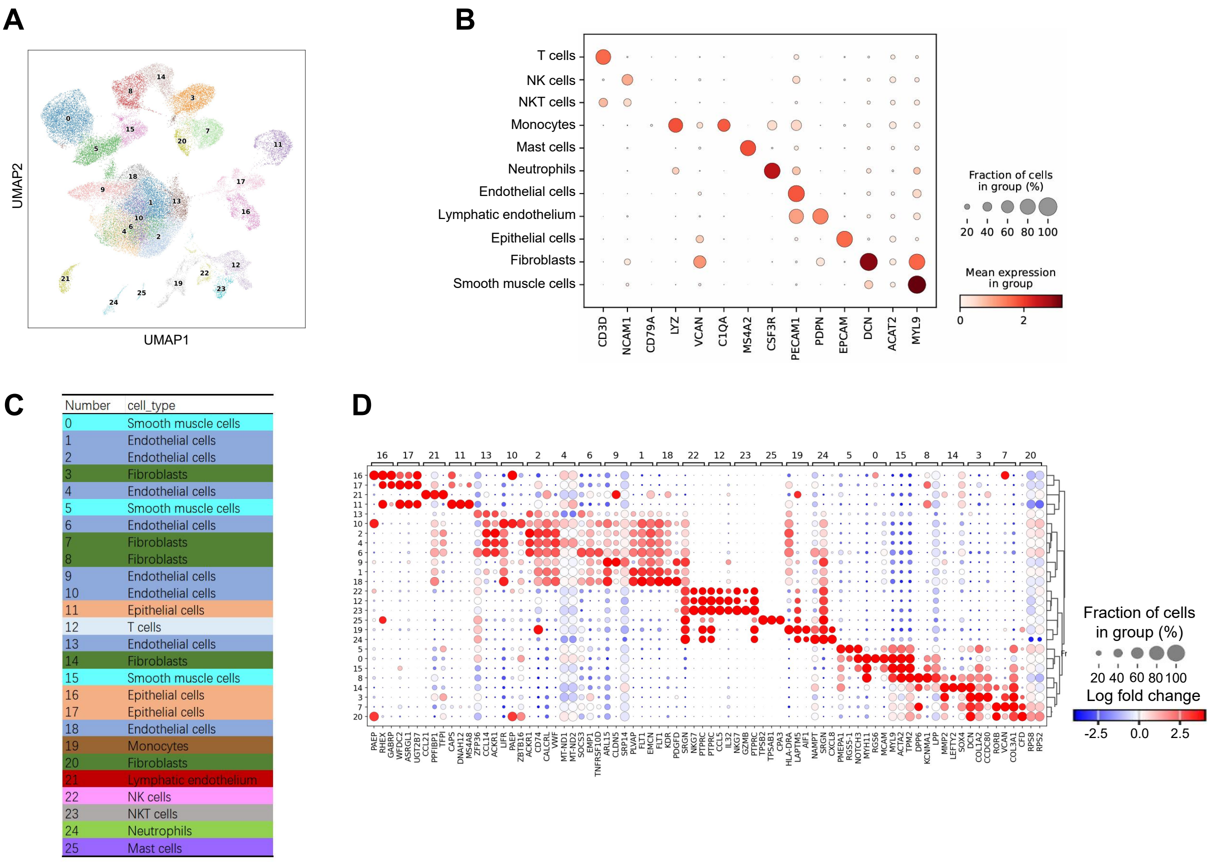

Supplementary Figure S7. A) The distribution of 26 main cell type clusters in six samples (NC, n=3; AM, n=3) from patients by UMAP plots. **B)** Dot plot for cell-type-specific signature genes. **C)** Annotation of 11 major cell clusters in six samples. **D)** Expression of three typical marker genes of each cluster. NC: negative control group; AM: adenomyosis group.

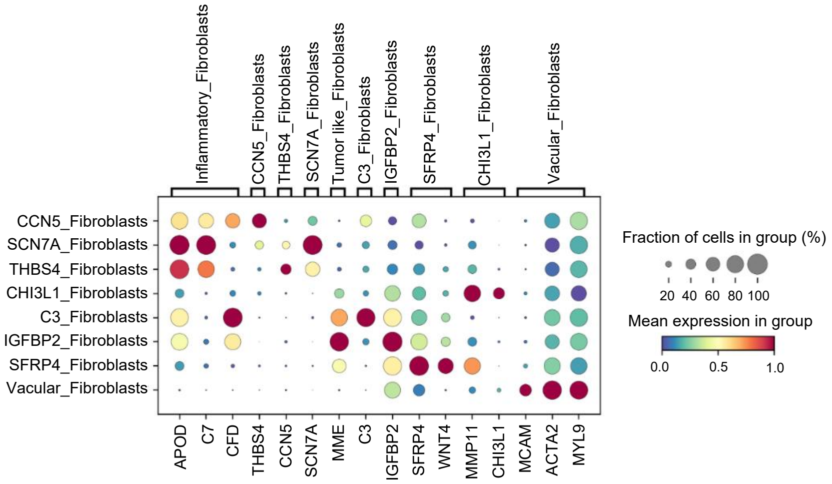

Supplementary Figure S8. Expression of typical marker genes of each fibroblast subcluster.

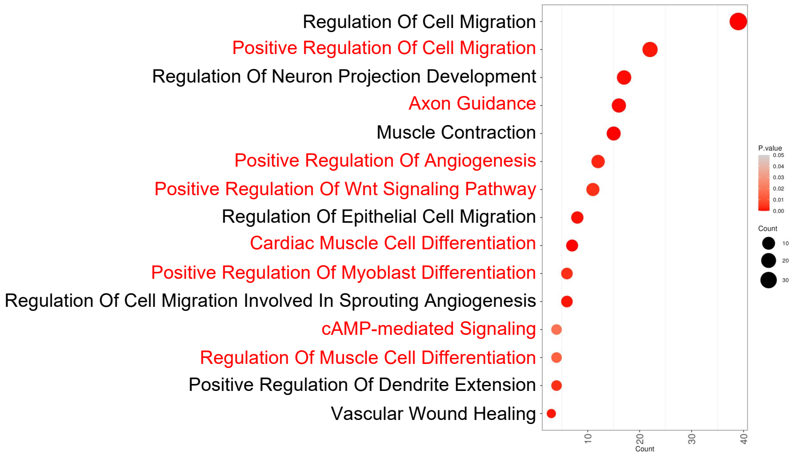

Supplementary Figure S9. GO (Gene Ontology) analysis of the Vascular_Fibroblast cluster

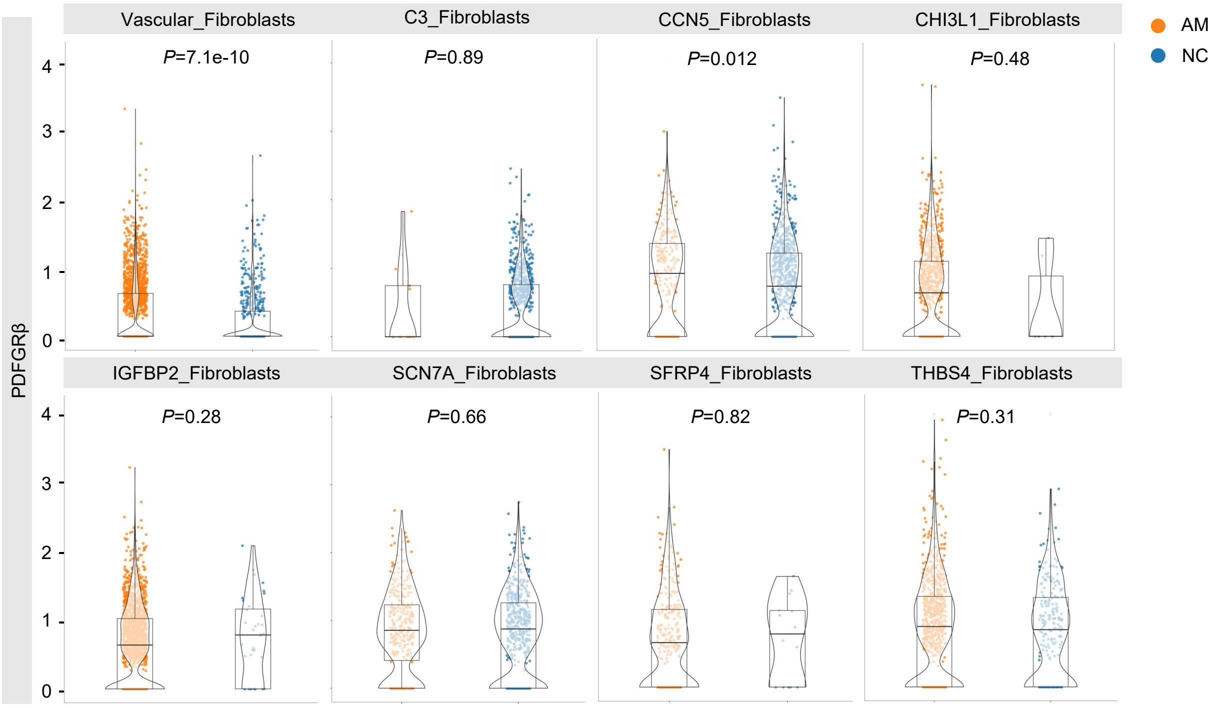

Supplementary Figure S10. The expression level of *PDGFRβ* in the eight fibroblast subclusters in AM and NC. n=3 patients in each group. NC: negative control group, AM: adenomyosis group.

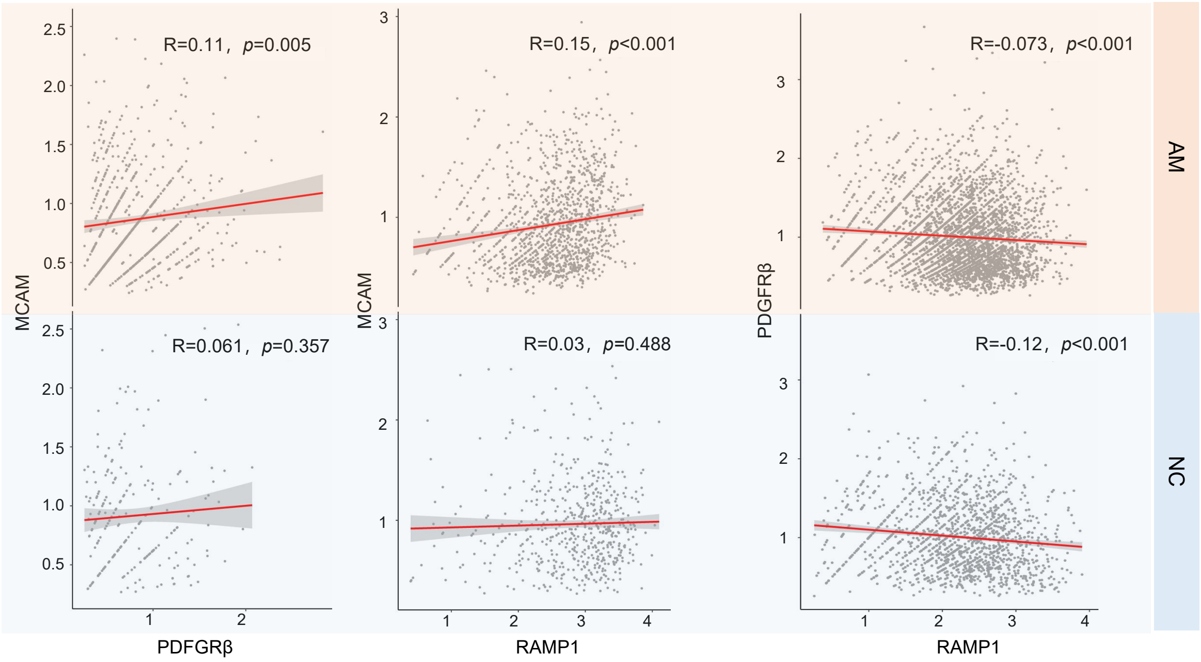

Supplementary Figure S11. Correlation analysis of *MCAM*, *PDGFRβ*, and *RAMP1* in the fibroblast cluster in AM and NC groups. n=3 patients per group. *MCAM*: Melanoma Cell Adhesion Molecule, gene name of CD146; *PDRFRβ*: Platelet Derived Growth Factor Receptor Beta, gene name of CD140b; *RAMP1*: Receptor Activity-Modifying Protein 1. NC: negative control group, AM: adenomyosis group.

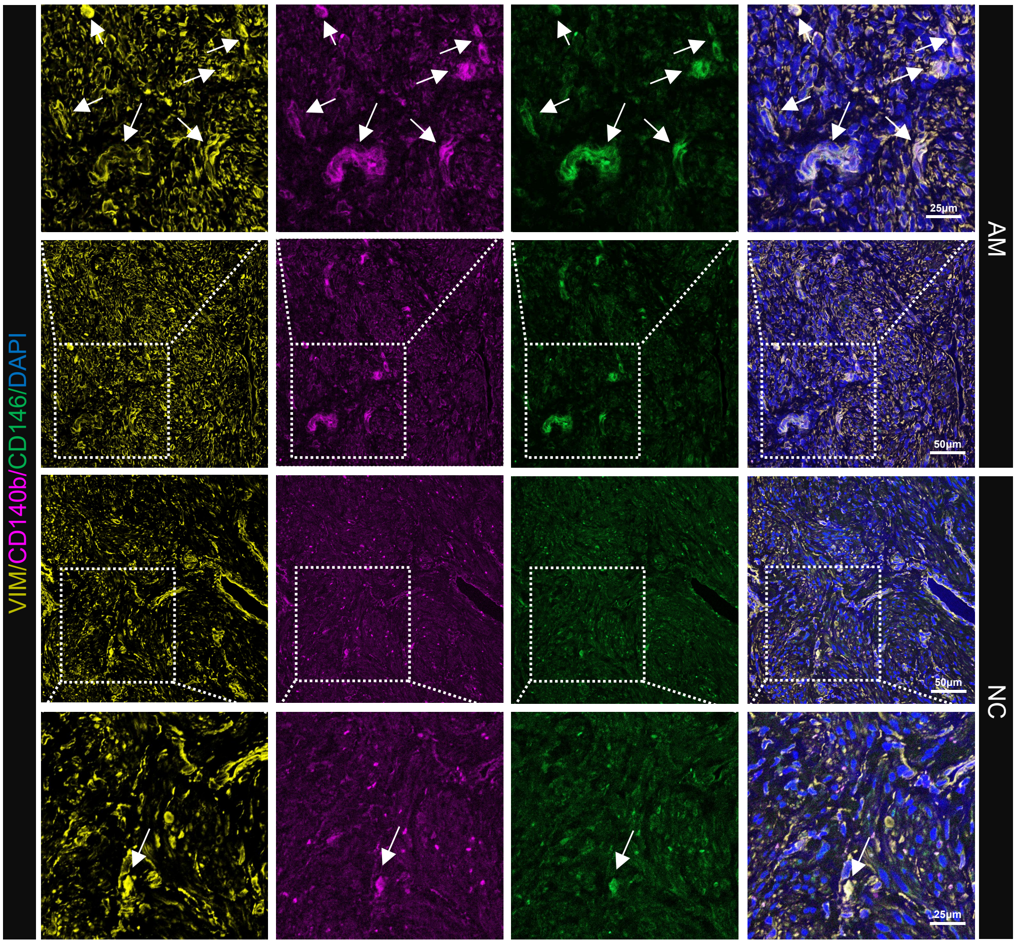

Supplementary Figure S12. Representative images of multiple IF staining of Vimentin (a marker for fibroblasts, yellow), CD140b (violet), CD146 (green), and nucleus (DAPI, Blue) in the myometrial tissue from AM and NC. n = 5 per group. Scale bar, top, 25µm; bottom, 25µm; middle, 50µm. AM: adenomyosis group; NC: negative control group. The arrows show the cells co-expressing Vimentin, CD140b and CD146.

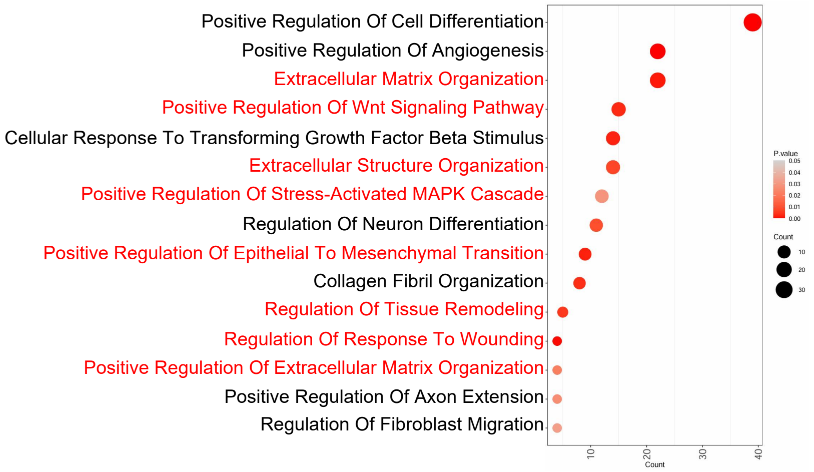

Supplementary Figure S13. GO (Gene Ontology) analysis of the CHI3L1_fibroblast cluster

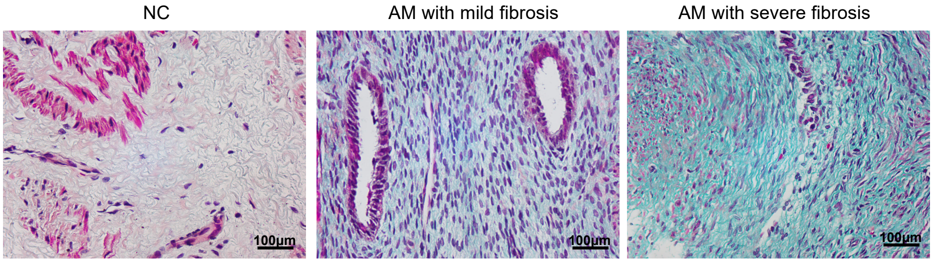

Supplementary Figure S14. Representative images of Masson-stained images in the myometrium from NC and AM. n=5 per group. Scale bar, 100μm; NC: negative control group; AM: adenomyosis group

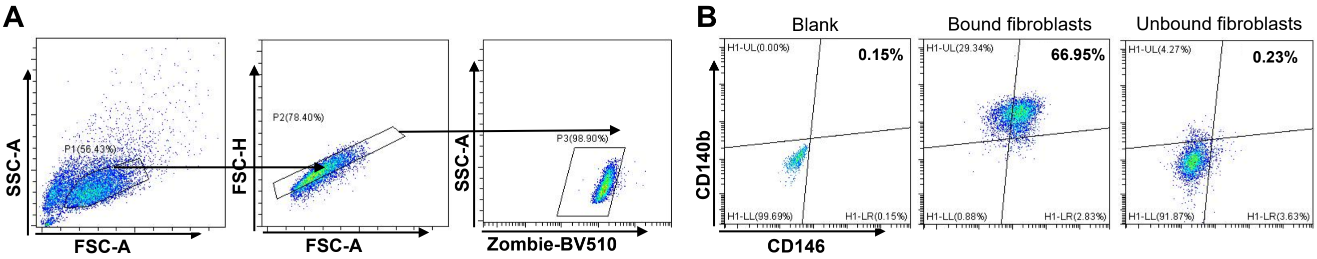

Supplementary Figure S15. **A)** Representative image of flow cytometry (FCM) gating strategy for fibroblasts. **B)** Representative image of AMDFs sorting efficiency using magnetic beads. AMDFs: Adenomyosis ectopic lesion-derived fibroblasts. FCM was performed three times on three independent samples from patients.

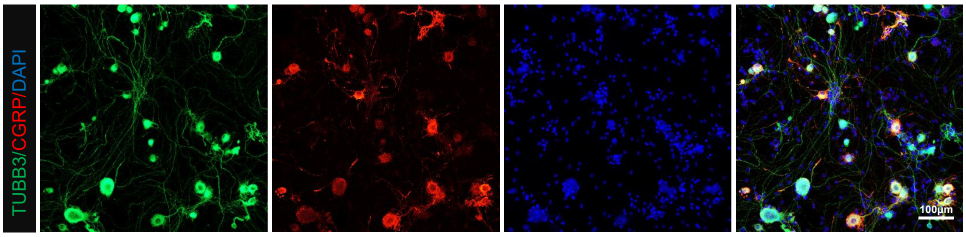

Supplementary Figure S16. Confirmation of primary rat DRG using IF (Green for TUBB3 and red for CGRP). Scale bar, 100 μm. The verification of DRG was performed at least three times before used in the experiment.

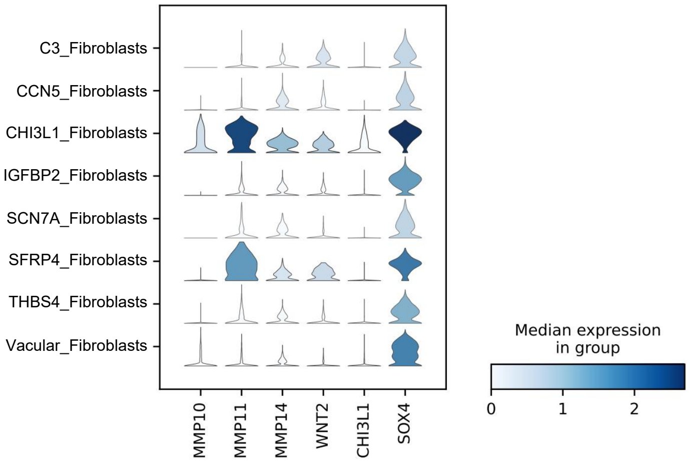

Supplementary Figure S17. Violin plots depict expression levels of *MMP10*, *MMP11*, *MMP14*, *WNT2*, *CHI3L1*, *SOX4* in eight fibroblast subclusters.

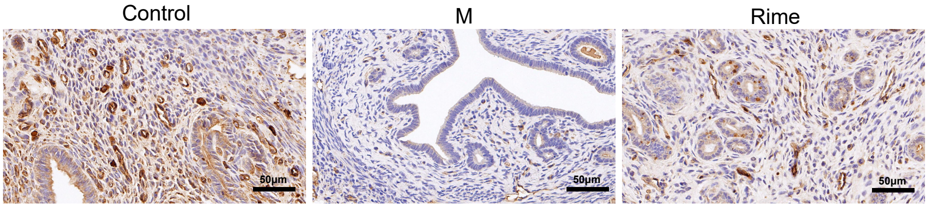

Supplementary Figure S18. Representative images of IHC staining of uteri from mice with CD31 in Control, M, and Rime groups. n = 8 per group. Scale bar, 50μm. Control: control group of mice; M: mice with induced adenomyosis; Rime: mice with induced adenomyosis and rimegepant treatment.

Table S1

The number of embryos and miscarriages per mouse following different treatments in fertility assessment

		Left uteri		Right uteri	
Group	Number	Live embryos	Aborted embryos	Live embryos	Aborted embryos
Control	1)	6	0	6	0
	2)	0	2	0	3
	3)	9	0	3	0
	4)	6	0	8	0
	5)	4	0	5	0
	6)	4	0	5	0
	7)	7	0	7	0
	8)	9	0	6	0
	9)	0	0	0	0
	10)	0	0	0	0
M	1)	0	0	0	0
	2)	0	0	0	0
	3)	0	0	0	0
	4)	0	0	3	0
	5)	0	0	2	0
	6)	0	0	0	0
	7)	0	0	0	0
	8)	0	0	0	0
	9)	0	0	5	0
	10)	0	2	0	6
	11)	3	0	3	2
	12)	3	2	3	2
	13)	1	2	2	2
	14)	1	0	4	0
	15)	0	0	0	0
	16)	3	1	3	1
	17)	0	0	0	0
	18)	4	0	3	1
	19)	0	0	0	0
	20)	0	0	0	0
	21)	0	0	0	0
	22)	0	0	0	0
	23)	0	0	0	0
	24)	0	0	0	0
	25)	0	0	0	0
	26)	0	0	0	0
	27)	1	3	0	1
Rime	1)	5	0	2	0
	2)	4	0	0	0
	3)	1	1	5	0
	4)	1	0	1	1
	5)	2	1	2	1
	6)	0	0	0	0
	7)	2	0	0	0
	8)	3	0	3	0
	9)	0	0	2	0
	10)	0	0	0	3
	11)	3	0	3	0
	12)	0	0	2	0
	13)	0	0	0	0
	14)	0	0	0	0
	15)	0	0	0	0
	16)	7	0	1	0
	17)	3	1	3	1
	18)	4	0	5	0
	19)	0	0	0	0
	20)	0	0	0	2
	21)	0	0	0	1
	22)	0	0	0	0

Control: n=10; M: mice with induced adenomyosis, n=27; Rime: mice with induced adenomyosis and rimegepant treatment, n=22.

Table S2

Clinical characteristics of adenomyosis and control patients in this study

Number	Pathologic diagnoses	Age	Dysmenorrhea	Procedure	Procedure of menstrual cycle	Methods
AM-01	Adenomyosis	41	Severe	LH	Proliferative phase	Sc-RNA sequencing
AM-02	Adenomyosis	35	Severe	RAL	Proliferative phase	Sc-RNA sequencing
AM-03	Adenomyosis	33	Severe	RAL	Secretory phase	Sc-RNA sequencing
AM-04	Adenomyosis	41	Moderate	RAL	Secretory phase	Validation
AM-05	Adenomyosis	36	Severe	RAL	Proliferative phase	Validation
AM-06	Adenomyosis	39	Severe	RAL	Proliferative phase	Validation
AM-07	Adenomyosis	42	Severe	RAL	Proliferative phase	Validation
AM-08	Adenomyosis	43	Moderate	TAH	Secretory phase	Validation
AM-09	Adenomyosis	32	Severe	RAL	Secretory phase	Validation
AM-10	Adenomyosis	40	Severe	RAL	Proliferative phase	Validation
AM-11	Adenomyosis	41	Severe	RAL	Secretory phase	Validation
AM-12	Adenomyosis	33	Severe	RAL	Proliferative phase	Validation, Cell Extraction
AM-13	Adenomyosis	52	Severe	RAL	Secretory phase	Validation
AM-14	Adenomyosis	40	Severe	RAL	Secretory phase	Validation, Cell Extraction
AM-15	Adenomyosis	47	Severe	TAH	Secretory phase	Validation
AM-16	Adenomyosis	47	Severe	TAH	Secretory phase	Validation
AM-17	Adenomyosis	46	Severe	LH	Secretory phase	Validation
AM-18	Adenomyosis	51	Severe	LH	Secretory phase	Validation
AM-19	Adenomyosis	37	Moderate	RAL	Secretory phase	Validation
AM-20	Adenomyosis	35	Mild	RAL	Proliferative phase	Validation
AM-21	Adenomyosis	47	Severe	TAH	Proliferative phase	Validation
AM-22	Adenomyosis	38	Severe	RAL	Proliferative phase	Validation, Cell Extraction
AM-23	Adenomyosis	40	Severe	RAL	Proliferative phase	Validation
NC-01	Cervical cancer	43	None	LH	Secretory phase	Sc-RNA sequencing
NC-02	Cervical cancer	36	None	LH	Secretory phase	Sc-RNA sequencing
NC-03	Hysteromyoma	45	None	LH	Proliferative phase	Sc-RNA sequencing
NC-04	Hysteromyoma	53	None	TAH	Secretory phase	Validation
NC-05	Hysteromyoma	48	Mild	LH	Secretory phase	Validation
NC-06	Hysteromyoma	47	None	TAH	Secretory phase	Validation
NC-07	Hysteromyoma	49	None	TAH	Proliferative phase	Validation
NC-08	Hysteromyoma	53	None	LH	Proliferative phase	Validation
NC-09	Hysteromyoma	43	None	LH	Secretory phase	Validation
NC-10	Hysteromyoma	49	None	TAH	Secretory phase	Validation
NC-11	Hysteromyoma	50	Mild	LH	Proliferative phase	Validation
NC-12	Cervical cancer	48	None	LH	Proliferative phase	Validation
NC-13	Hysteromyoma	44	None	LH	Proliferative phase	Validation
NC-14	Hysteromyoma	49	None	LH	Proliferative phase	Validation
NC-15	Hysteromyoma	49	None	LH	Proliferative phase	Validation
NC-16	Hysteromyoma	51	None	LH	Proliferative phase	Validation
NC-17	Hysteromyoma	46	None	LH	Proliferative phase	Validation
NC-18	Hysteromyoma	53	None	TAH	Proliferative phase	Validation
NC-19	Hysteromyoma	53	None	LH	Proliferative phase	Validation
NC-20	Hysteromyoma	48	None	LH	Proliferative phase	Validation
NC-21	Hysteromyoma	51	None	TAH	Secretory phase	Validation
NC-22	Hysteromyoma	42	None	TAH	Proliferative phase	Validation
NC-23	Hysteromyoma	33	None	LH	Secretory phase	Validation

Abbreviation: TAH, Transabdominal hysterectomy; LH, laparoscopic hysterectomy; RAL, resection of adenomyosis lesions.

Table S3

Primer name	Primer sequence (5′to3′)
hGAPDH-F	GCACCGTCAAGGCTGAGAAC
hGAPDH-R	TGGTGAAGACGCCAGTGGA
hACTA2-F	CTATGCCTCTGGACGCACAACT
hACTA2-R	CAGATCCAGACGCATGATGGCA
hFN-F	AGGAAGCCGAGGTTTTAACTG
hFN-R	AGGACGCTCATAAGTGTCACC
hCOL1A1-F	TACCACTGCAAGAACAGCGT
hCOL1A1-R	AATCACTGTCTTGCCCCAGG
hNGF-F	TTCCCTTGACACTGCCCTTC
hNGF-R	GAATTCGCCCCTGTGGAAGA
hPGP9.5-F	CGTGGATGGCCACCTCTATG
hPGP9.5-R	GAAGCGGACTTCTCCTTGCT
hRAMP1-F	CACTGAGAAATCCGGCCCAT
hRAMP1-R	CTACCTGGAACTGGGTGAGG
hCHI3L1-F	TGGGTCTCAAAGATTTTCCAAGA
hCHI3L1-R	TCGGCCTTCATTTCCTTGATT
hIGFBP2-F	GACAATGGCGATGACCACTCA
hIGFBP2-R	CAGCTCCTTCATACCCGACTT
hSFRP4-F	ACGAGCTGCCTGTCTATGAC
hSFRP4-R	TGTCTGGTGTGATGTCTATCCAC
hTHBS4-F	GCACCTTCCCATCGTTCTTCAG
hTHBS4-R	GTTCAGCCACCATCTTCGGTC
hPDGFRβ-F	CCTGCAATGTGACGGAGAGT
hPDGFRβ-R	GGTGCGGTTGTCTTTGAACC
hMCAM-F	GACGAGCGCATCTTCTTGTG
hMCAM-R	GGGGGTTGACCTGGATGTTT
hSOX4-F	CCAAATCTTTTGGGGACTTTT
hSOX4-R	CTGGCCCCTCAACTCCTC
hWNT2-F	CCAGCCTTTTGGCAGGGTC
hWNT2-R	GCATGTCCTGAGAGTCCATG
hMMP10-F	ATCTGAGATGCCAGCCAAGT
hMMP10-R	AGGGTTCCAGTGGGATCTTC
hMMP11-F	TCCCCAAGACTCACCGAGAA
hMMP11-R	GTAGAAGGCGGACATCAGGG
hMMP14-F	CATCTGTGACGGGAACTTTGA
hMMP14-R	GGCAGTGTTGATGGACGCA

mGAPDH-F	CTCAGGAGAGTGTTTCCTCGTC
mGAPDH-R	ATGGGCTTCCCGTTGATGAC
mACTA2-F	CCCAGACATCAGGGAGTAATGG
mACTA2-R	TCTATCGGATACTTCAGCGTCA
mFN-F	GCCGTTAGATGTGCAAGCTG
mFN-R	TGCTGAAGCTGAGAACTAGGC
mCOL1A1-F	GCTTCCCAGATGTCCTATGGC
mCOL1A1-R	CATTGGACCTGAACCGCCAG
mPGP9.5-F	CCCCGAGATGCTGAACAAAGT
mPGP9.5-R	GGAGTTTCCGATGGTCTGCTT
mNGF-F	GCGTTTTTGATCGGCGTACA
mNGF-R	AGGGCTGTGTCAAGGGAATG
mRAMP1-F	GAGACTATTGGGAAGACGCTATG
mRAMP1-R	CTCCTCCAGACCACCAGTG
